# Resistance of mRNAs with AUG-proximal nonsense mutations to nonsense-mediated decay reflects variables of mRNA structure and translational activity

**DOI:** 10.1093/nar/gkv588

**Published:** 2015-06-11

**Authors:** Francisco J.C. Pereira, Alexandre Teixeira, Jian Kong, Cristina Barbosa, Ana Luísa Silva, Ana Marques-Ramos, Stephen A. Liebhaber, Luísa Romão

**Affiliations:** 1 Departamento de Genética Humana, Instituto Nacional de Saúde Doutor Ricardo Jorge, 1649-016 Lisboa, Portugal; 2 Centro de Investigação em Genética Molecular Humana, Faculdade de Ciências Médicas, Universidade Nova de Lisboa, 1349-008 Lisboa, Portugal; 3 Departments of Genetics and Medicine, University of Pennsylvania, Philadelphia, PA 19104, USA; 4 BioISI - Biosystems & Integrative Sciences Institute, Faculdade de Ciências, Universidade de Lisboa, 1749-016 Lisboa, Portugal

## Abstract

Nonsense-mediated mRNA decay (NMD) is a surveillance pathway that recognizes and selectively degrades mRNAs carrying premature termination codons (PTCs). The level of sensitivity of a PTC-containing mRNA to NMD is multifactorial. We have previously shown that human β-globin mRNAs carrying PTCs in close proximity to the translation initiation AUG codon escape NMD. This was called the ‘AUG-proximity effect’. The present analysis of nonsense codons in the human α-globin mRNA illustrates that the determinants of the AUG-proximity effect are in fact quite complex, reflecting the ability of the ribosome to re-initiate translation 3′ to the PTC and the specific sequence and secondary structure of the translated ORF. These data support a model in which the time taken to translate the short ORF, impacted by distance, sequence, and structure, not only modulates translation re-initiation, but also impacts on the exact boundary of AUG-proximity protection from NMD.

## INTRODUCTION

The classical model of nonsense-mediated mRNA decay (NMD) in mammalian cells stipulates that the relationship of a nonsense codon to exon–exon junctions in a PolII transcript dictates whether it will be recognized as ‘premature’ and trigger rapid decay. This decay, when it occurs, is triggered by an interaction of the translation termination complex at the stop codon with a retained exon junction complex (EJC) on the mRNA (1–6). These protein interactions appear to be critical to the discrimination of a premature translation termination event from a normal one (1–3,5,6). The EJC is deposited 20–24 nucleotides (nts) upstream of the exon–exon junction(s) during splicing and remains associated with the mRNA during its transport to the cytoplasm (1–3). Translating ribosomes subsequently displace EJCs from the open reading frame (ORF) during the pioneer round of translation. If a stop codon is located more than 50–54 nts upstream of at least one exon–exon junction, the leading edge of the elongating ribosome will fail to displace it. In this case, when the ribosome reaches the termination codon, the translation eukaryotic release factors eRF1 and eRF3 at the stop codon interact *in cis* with the retained EJC(s) *via* bridging interactions between the release complex associated proteins, UPF1 and SMG-1 and the EJC-associated factors, UPF2-UPF3 (7,8). This bridging interaction triggers accelerated decay (i.e. NMD) of the nonsense-containing mRNA through the recruitment of additional factors (9–19).

In addition to the EJC-dependent NMD model, an EJC-independent NMD pathway postulates that identification of a stop codon as ‘premature’ depends on the physical distance between the stop codon and the cytoplasmic poly(A)-binding protein 1 (PABPC1) bound to the poly(A) tail (20–25). This ‘faux 3′ UTR’ model proposes that PABPC1 and UPF1 compete for interaction with eRF3 at the site of translational termination: if PABPC1 is in close proximity to a stop codon, it interacts with the termination complex, stimulates translation termination (26), and represses NMD; alternatively, when the interaction of PABPC1 with the termination complex is reduced, for example due to a long 3′ untranslated region (3′ UTR), UPF1 interacts with eRF3 and triggers NMD (20–25). Recent studies that map UPF1 binding throughout the mRNA (5′ UTRs, coding regions and 3′ UTR) (27–29) irrespective of NMD (28) seem to challenge this mechanistic model of NMD. Nevertheless, elongating ribosomes displace UPF1 from coding sequences causing its enrichment in 3′ UTRs (28); thus, transcripts with long 3′ UTRs might increase the probability that UPF1 will outcompete PABPC1 for release factor binding and trigger NMD.

Consistent with the faux 3′ UTR model of NMD is the fact that endogenous NMD substrates are enriched in mRNAs containing long 3′ UTRs (30–33). This model is also supported by the observation that artificially tethering PABPC1 in close proximity to a premature termination codon (PTC) can inhibit NMD through a mechanism that involves its eRF3-interacting C-terminal domain (21–24,34). However, recent data have shown that interaction of PABPC1 with eRF3 is not strictly necessary for the tethered PABPC1 to suppress NMD (35), as NMD suppression may also be mediated *via* PABPC1 interaction with the eukaryotic initiation factor 4G (eIF4G) (36,37). Furthermore, it has been suggested that a key NMD determinant might be the efficiency of ribosome release at the PTC (38), which is an event where UPF1 seems to have a role (39). These and other observations (reviewed in reference 38) reinforce the conclusion that the mechanisms that dictate NMD strength are complex and not well defined.

The pivotal role that PABPC1 plays in NMD suppression when in close proximity to a stop codon can also be highlighted by the ‘AUG-proximity effect’. Studies from our laboratory have shown that human β-globin (hβ-globin) mRNAs containing nonsense mutations early in exon 1 accumulate to levels similar to those of wild-type (WT) β-globin transcripts (40). This resistance to NMD is erythroid- and promoter-independent, and does not reflect translation re-initiation, abnormal RNA splicing, or impaired translation (41). Instead, the observed NMD-resistance reflects the close proximity of the nonsense codon to the translation initiation codon (41). This was called the ‘AUG-proximity effect’ (21). Consistent with the repressive impact of PABPC1 on NMD (see above) (20–24) our mechanistic studies revealed that the AUG-proximity effect results the juxtaposition of PABPC1 with the AUG-proximal PTC as a consequence of mRNA circularization and the inherent nature of the short ORF translation process (21,34).

In the present report, we carry out a detailed comparison of the AUG-proximity effect on NMD of the human α- and β-globin mRNAs. While the data support a generality of the AUG-proximity effect on NMD, this detailed comparison also highlights variables of mRNA sequence and structure that factor into this pathway of NMD resistance. The impact of these variables appears to reflect the time taken for the 80S ribosome to translate the short ORF prior to encountering the PTC.

## MATERIALS AND METHODS

### Construction of expression vectors

The 1677-bp EcoRI/RcaI fragment containing the whole wild-type α2-globin gene was obtained from plasmid pTet-αWT (42) and sub-cloned into EcoRI/BspLU11I sites of the pTRE2pur vector (BD Biosciences), originating the pTRE-αWT plasmid. Constructs α4 (CCU→UAG), α7 (AAU→UAG), α10 (GUC→UAG), α12 (GCC→UAG), α14 (UGG→UAG), α14/40 (UGG→UAG, AAG→UAG), α16 (AAG→UAA), α19 (GCG→UAG), α21 (GCU→UAG), α23 (GAG→UAG), α25 (GGU→UAG), α27 (GAG→UAG), α30 (GAG→UAG), α32 (AUG→UAG), α36 (UUC→UAG), α40 (AAG→UAG), α45 (CAC→UAG), α50 (CAC→UAG), α55 (GUU→UAG), α60 (AAG→UAG), α65 (CGC→UAG), α70 (GUG→UAG), α73 (GUG→UAG), α76 (AUG→UAG), α78 (AAC→UAG), α80 (CUG→UAG), α82 (GCC→UAG), α84 (AGC→UAG), α86 (CUG→UAG), α93 (GUG→UAA), α101 (CUA→UAG) and α116 (GAG→UAG), carrying the denoted nonsense mutations at codon positions indicated by the respective number, were created by site-directed mutagenesis, as recommended by the kit manufacturer (QuikChange Site-Directed Mutagenesis Kit; Stratagene), with mutagenic primers #1-#62 (Supplementary Table S1), using the plasmid pTRE-αWT as DNA template. Potential re-initiation sites in α-globin codons 32 and 76 were sequentially mutated, from AUG (methionine: Met) to ACG (threonine: Thr), in *cis* by site-directed mutagenesis with primers #63-#66, using pTRE-αWT, α14, α27 or α40 as templates, to create the constructs αWT.32–76Met→Thr, α14.32–76Met→Thr, α27.32–76Met→Thr and α40.32–76Met→Thr.

The αWT-pseudoknot (pk), α25-pk, α27-pk and α40-pk gene variants were prepared by replacing the first 19 codons of the native α-globin ORF by a 19-codon sequence resulting in a pseudoknot structure in the mRNA (43), by overlap-extension polymerase chain reaction (PCR) with overlapping primers #67 and #68 (Supplementary Table S1) and the αWT, α25, α27 or α40 genes as DNA templates. The αWT-(CAA)_26_ and α40-(CAA)_26_ gene variants were obtained by replacing the first 26 codons of the α-globin ORF with 26 consecutive ‘CAA’ repeats, also by overlap-extension PCR, with overlapping primers #69 and #70 and the αWT or α40 genes as DNA templates. The α27-(CAA)_26_ gene was created with overlapping primers #71 and #72 and the αWT-(CAA)_26_ gene as DNA template. The αWT-(CAA)_26–_32–76Met→Thr, α27-(CAA)_26–_32–76Met→Thr and α40-(CAA)_26–_32–76Met→Thr gene variants were produced with overlapping primers #63 and #64 and the αWT-(CAA)_26_, α27-(CAA)_26_, αWT.32–76Met→Thr or α40-(CAA)_26–_32–76Met→Thr genes as DNA templates. For all the above mentioned α-globin gene variants prepared by overlapping PCR, flanking primers #73 and #74 were used and a 923-bp KpnI/ApaI fragment of each PCR product was cloned into the KpnI/ApaI sites of the pTRE-αWT plasmid.

The wild-type β-globin gene (βWT), as well as the previously described human β-globin variants β15 (UGG→UGA) and β39 (CAG→UAG) (41), were sub-cloned into the ClaI/BspLU11I sites of pTRE2pur vector (BD Biosciences) by PCR amplification of the 1806-bp ClaI/BspLU11I fragment, using primers with linkers for ClaI and BspLU11I (primers #75 and #76; Supplementary Table S1). The β-globin variants β23 (GUU→UAG), β25 (GGU→UAG) and β26 (GAG→UAG), carrying the denoted nonsense mutations at codon positions 23, 25 and 26, respectively, were created by site-directed mutagenesis using mutagenic primers #77-#82 and the construct βWT as DNA template. The βWT-pk, β23-pk and β39-pk gene variants were constructed by replacing the first 19 codons of a native β-globin ORF by a 19-codon sequence resulting in a pseudoknot structure in the mRNA (43), using the ExSite PCR-Based Mutagenesis Kit (Stratagene) as indicated by the manufacturer, with mutagenic primers #83 and #84, and the βWT and β39 genes as DNA templates, or primers #83 and #85 using the β23 gene as DNA template. The βWT-(CAA)_25_ and β39-(CAA)_25_ gene variants were obtained by replacing the first 25 codons of the native β-globin ORF with 25 consecutive ‘CAA’ repeats, by overlap-extension PCR with overlapping primers #86 and #87, and the βWT or β39 genes as DNA templates. The β26-(CAA)_25_ gene was prepared with overlapping primers #88 and #89, and the βWT-(CAA)_25_ gene as template. Flanking primers #73 and #90 were used and a 478-bp ClaI/BbrPI fragment of each PCR product was cloned into the ClaI/BbrPI sites of the βWT construct.

The hybrid genes 5′ UTR-β/αWT, 5′ UTR-β/α27, 5′ UTR-β/α40, βWT26/α, β26/α‘27’ and βWT26/α40 were obtained by replacing the 5′ untranslated region (5′ UTR) or the first 26 codons plus the 5′ UTR of the respective α-globin gene variants by the equivalent β-globin sequences. This was achieved by overlap-extension PCR with overlapping primers #91-#96 (Supplementary Table S1) and flanking primers #73 and #74, and the αWT, α27, α40, βWT or β26 genes as DNA templates. Additionally, 5′ UTR-α/βWT26/α, 5′ UTR-α/β26/α‘27’ and 5′ UTR-α/βWT26/α40 genes were shaped by replacing the 5′ UTR of βWT26/α, β26/α‘27’ and βWT26/α40, respectively, with the native 5′ UTR of α-globin. Overlapping primers #97 and #98 were used together with flanking primers #73 and #74 on αWT, βWT26/α, β26/α‘27’ and βWT26/α40 template genes. A 1094-bp XbaI/BbrPI fragment of each PCR product was cloned into the XbaI/BbrPI sites of the αWT construct. The hybrid genes 5′ UTR-α/βWT, 5′ UTR-α/β26, 5′ UTR-α/β39, αWT27/β, α27/β‘26’ and αWT27/β39 were produced reciprocally to β/α-globin hybrid genes, with overlapping primers #97-#102 and flanking primers #73 and #90, and the βWT, β26, β39, αWT or α27 genes as DNA templates. Furthermore, the hybrid genes 5′ UTR-β/αWT27/β, 5′ UTR-β/α27/β‘26’ and 5′ UTR-β/αWT27/β39 were obtained using overlapping primers #91 and #92, flanking primers #73 and #90, and the βWT, αWT27/β, α27/β and αWT27/β39 genes as templates. The 1110-bp XbaI/BbrPI fragment of each PCR product was inserted into the XbaI/BbrPI sites of the βWT construct.

The 514-bp XhoI/HindIII fragment containing the *P*_hCMV*-1_ tetracycline (tet)-responsive promoter was obtained from the pTRE2pur vector (BD Biosciences) and sub-cloned into XhoI/HindIII sites of the pGL2-Enhancer vector (Promega), which contains the firefly luciferase gene, and into XhoI/HindIII sites of the pGL4.70[*hRluc*] vector (Promega), which contains the *Renilla* luciferase gene, originating the pGL2TRE and the pGL4TRE plasmids, respectively.

The α-globin/luciferase (Luc) hybrid genes, α14/Luc, α27/Luc, α27-(CAA)_26_/Luc and α27-pk/Luc, were obtained by replacing the α-globin sequence downstream to codon 32 with the firefly luciferase ORF, by overlap-extension PCR with overlapping primers #103 and #104 (Supplementary Table S1) and the α14, α27, α27-(CAA)_26_ and α27-pk or pGL2TRE constructs as DNA templates. The respective positive and negative controls for luciferase activity were created with overlapping primers #105-#108 and the α14/Luc or α27/Luc genes as DNA templates. Flanking primers #73 and #109 were used and a 752-bp XhoI/XbaI fragment of each PCR product was cloned into the XhoI/XbaI sites of the pGL2TRE plasmid.

The β-globin/Luc hybrid genes, β15/Luc, β23/Luc and β26/Luc, were prepared by replacing the β-globin sequence downstream to codon 55 with the firefly luciferase ORF, by overlap-extension PCR with overlapping primers #110 and #111 (Supplementary Table S1) and the β15, β23, β26 or pGL2TRE constructs as DNA templates. The respective positive and negative controls for luciferase activity were produced with overlapping primers #112-#115 and the β15/Luc, β23/Luc or β26/Luc genes as DNA templates. Flanking primers #73 and #109 were used and a 906-bp XhoI/XbaI fragment of each PCR product was cloned into the XhoI/XbaI sites of the pGL2TRE plasmid.

### Cell culture and transfections

Mouse erythroleukemia (MEL) cells stably expressing the tet transactivator (MEL/tTA) (42) were used for conditional expression of human α-globin genes (previously cloned into the pTRE2pur vector). For transient transfections, MEL/tTA cells were split 1 day before transfection and cultured in minimal essential medium (MEM) supplemented with 10% (v/v) fetal bovine serum, and 100 ng/ml tetracycline (tet). Cells were transfected with 2 μg of pTRE-αWT, or each variant, as previously described. Then, cells were split into 60-mm-diameter dishes, and pulsed with α-globin mRNA for 4 h by growth in tet(-) media. Following this period, transcription from the plasmid was blocked by the addition of tet to the media. Cells from each culture dish were harvested in different time points for further analyses.

HeLa cells, stably expressing the tet transactivator (HeLa/tTA) (42), were grown in Dulbecco's modified Eagle's medium (DMEM) supplemented with 10% (v/v) fetal bovine serum. Transient transfections were performed using Lipofectamine 2000 Transfection Reagent (Invitrogen), following the manufacturer's instructions, in 35-mm plates. For genes cloned into the pTRE2pur vector, 150 ng of the test construct DNA were cotransfected with 1850 ng of pEGFP plasmid DNA (BD Biosciences) as a control for monitoring transfection efficiency, and cells were harvested after a 20 h transcription pulse. For gene constructs cloned into the pGL2TRE plasmid, 2 μg of the test construct DNA were cotransfected with 50 ng of the pGL4TRE plasmid as a control for luminescence, and cells were harvested after a 16 h transcription pulse. When appropriated, transcription was blocked by the addition of tet or 50 μg/ml adenosine analogue 5,6-dichloro-1–3-D-ribofuranosylbenzimidazole (DRB) to the media and cells were harvested in different time points after treatment.

### Transfection of siRNA

Transient transfections of siRNAs were carried out using Lipofectamine 2000 reagent (Invitrogen) according to the manufacturer's instructions in 35-mm plates using 100 pmol of siRNA oligonucleotides and 4 μl of transfection reagent. Twenty-four hours later, cells were transfected again with 50–75 pmol of siRNAs, 150 ng of the test construct DNA and 1000 ng of pEGFP vector. After additional 24 h, cells were harvested for analysis of RNA and protein expression. When appropriated, HeLa cells were treated with 50 μg/ml DRB and RNA was thereafter extracted at different time points for further analysis. The siRNA oligonucleotides used for transfections [Luciferase (5′-AA-CGUACGCGGAAUACUUCGA-3′) and hUPF1 (5′-AA-GAUGCAGUUCCGCUCCAUU-3′)] were purchased as annealed, ready-to-use duplexes from Dharmacon.

### RNA isolation

Total RNA from transfected cells was prepared using the RNeasy mini kit (Qiagen) following the manufacturer's indications. RNA samples were treated with RNase-free DNase I (Ambion) and purified by phenol:chloroform extraction. Before further analyses, mRNA samples were tested by reverse-transcription (RT) followed by PCR (RT-PCR) to reject the hypothesis of activation of cryptic splicing pathway(s), with consequent alteration in mRNA sequence and possible circumvention of the premature termination codon. From all transcript species a single full-length product was amplified (data not shown), demonstrating that the studied nonsense transcripts present a normal splicing pattern.

### Ribonuclease protection assay (RPA)

The α-globin probe is a 174-bp fragment encompassing the 3′ part of exon 3 inserted into the polylinker region of pTRI-amp-18 (Ambion) and was generated by *in vitro* transcription, using a Maxiscript T7 or SP6 kit (Ambion), according to the manufacturer's standard protocol. The β-globin probe was produced using a Maxiscript SP6 kit (Ambion) and consists of a 170 bp fragment encompassing the last 20 bp of intron 2, the entire exon 3 coding region, and the first 21 bp of 3′ UTR, which was amplified by PCR and inserted into the polylinker region of pGEM3 (Promega). The puro^R^ probe was generated using a Maxiscript T7 kit (Ambion), comprises a 280 bp puromycin-resistance gene fragment cloned into pGEM3 and protects 197 nt of the puromycin-resistance mRNA. Samples were processed as described elsewhere (41,44).

### Western blot analysis

Protein lysates were resolved, according to standard protocols, in 10% SDS-PAGE, and transferred to polyvinylidene difluoride (PVDF) membranes (Bio-Rad). Membranes were probed using mouse monoclonal anti-α-tubulin (Roche) at 1:10000 dilution, and goat polyclonal anti-hUPF1 (Bethyl Labs) at 1:500 dilution. Detection was carried out using secondary peroxidase-conjugated anti-mouse IgG (Bio-Rad) or anti-goat IgG (Sigma) antibodies followed by chemiluminescence.

### Semi-quantitative RT-PCR

The reverse-transcription (RT) of 500 ng of RNA from HeLa cells cotransfected with pGL2TRE (or α- and β-globin/Luc hybrid variants; containing the firefly luciferase gene) and pGL4TRE (which contains the *Renilla* luciferase gene) was performed with Superscript II (Invitrogen), under conditions recommended by the manufacturer, using 2 pmol of reverse primers #109 and #116 (Supplementary Table S1) in a final volume of 10 μl. The PCR reactions for firefly and *Renilla* luciferases were executed in parallel at similar conditions: 1 μl of the RT product was amplified in a 25 μl reaction volume using 0.2 mM dNTP mixture, 1.5 mM MgCl_2_, 10 pmol of each primer (primers #109 and #117 for the firefly luciferase and primers #116 and #118 for *Renilla* luciferase), 0.75 U of GoTaq DNA Polymerase (Promega) and 1x Reaction Buffer (Promega). Thermocycler conditions were 95°C for 3 min, followed by 29 cycles of 95°C for 30 s, 58°C for 45 s and 72°C for 45 s, followed by a final extension of 72°C for 10 min. Samples were resolved by electrophoresis in 1% agarose gels stained with ethidium-bromide, which were then digitalized and densitometric analysis was performed using ImageQuant software (Molecular Dynamics).

### Reverse transcription-coupled quantitative PCR (RT-qPCR)

First-strand cDNA was synthesized from 2 μg of total RNA using the SuperScript II Reverse Transcriptase (Invitrogen) according to the manufacturer's instructions. Real-time PCR was performed with the ABI7000 Sequence Detection System (Applied Biosystems) using SYBR Green PCR Master Mix (Applied Biosystems). The relative expression levels of β-globin and α-globin mRNAs were normalized to the internal control puromycin-resistance mRNA in HeLa cells and calculated using the comparative C_T_ method (2^−ΔΔC^_T_) (45). The C_T_ values of variant β-globin and α-globin mRNAs amplicons were compared to the respective βWT, αWT counterpart or to βWT Luc siRNA, αWT Luc siRNA as indicated in figures and normalized with the reference amplicon C_T_ value. The amplification efficiencies of the β-globin, α-globin targets and puromycin-resistance reference amplicons were determined for each assay by dilution series. The forward and reverse primers for human β-globin mRNA were 5′-GTGGATCCTGAGAACTTCAGGCT-3′ and 5′-CAGCACACAGACCAGCACGT-3′ and for α-globin were 5′-CCCGGTCAACTTCAAGCTCC-3′ and 5′-CAGCACGGTGCTCACAGAAG-3′. In addition, we have used the following primers: 5′-CGCAA CCTCCCCTTCTACG-3′ and 5′-GGTGACGGTGAAGCCGAG-3′ for puromycin-resistance mRNA. The following cycling parameters were used: 10 min at 95ºC and 40 cycles of 15 s at 95ºC and 1 min at 60ºC. Technical triplicates from three independent experiments were assessed in all cases. To check for DNA contamination, quantitative PCR without reverse transcription was also performed.

### Luminometry assays

HeLa cells were lysed with Passive Lysis Buffer (Promega) and luminescence was measured in a Lucy 2 luminometer (Anthos Labtec) with the Dual-Luciferase Reporter Assay System (Promega), according to the manufacturer's standard protocol, using automatic injectors.

## RESULTS

### The AUG-proximity boundaries that dictate NMD resistance differ between human β- and α-globin mRNAs

We have previously reported that human β-globin mRNAs carrying nonsense mutations in proximity to the initiation AUG are resistant to NMD and are expressed at levels approaching those of the wild-type β-globin (βWT) mRNA (40,41). To further characterize this ‘AUG-proximity effect’ and to define its parameters, we compared the boundary of this effect in human β-globin and α-globin mRNAs.

**Figure 1.**
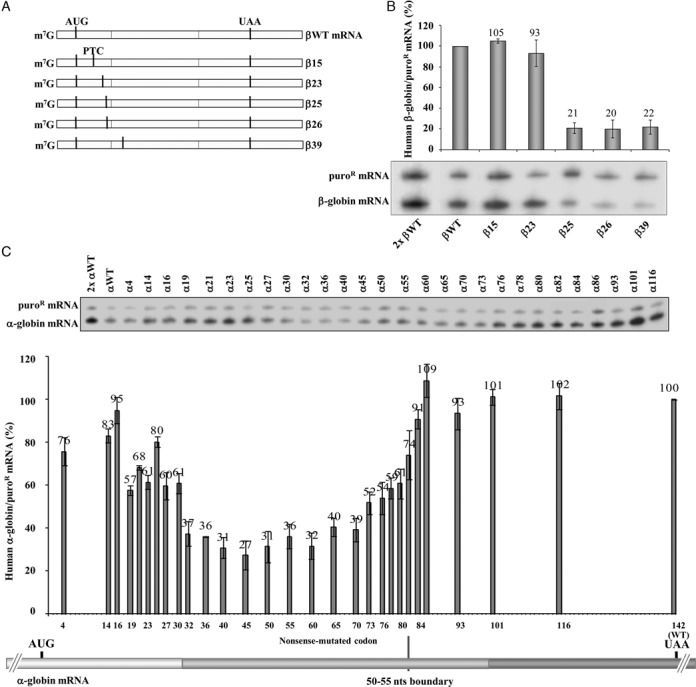
Fine-mapping reveals differences in the boundary of the AUG-proximity effect for NMD resistance in the human β- and α-globin mRNAs. (**A**) Schematic representation of the studied human β-globin mRNAs. Vertical lines represent translation initiation (AUG) or termination [native (UAA) or premature (PTC)] codons. The designation of each transcript is indicated to the right. (**B**) Representative ribonuclease-protection assay (RPA) using RNA isolated from HeLa cells transiently transfected with the constructs specified beneath each lane [the plasmid also contains the puromycin-resistance (puro^R^) gene]. The identification of β-globin and the puromycin-resistance (puro^R^) protected fragments is indicated to the left of the autoradiograph. Resulting levels of β-globin mRNA quantified relatively to the puro^R^ mRNA, and normalized to the expression level of the wild-type mRNA (βWT), are plotted above each respective lane. The average values and standard deviations from three independent experiments are shown. A 2-fold RNA sample (2x βWT) from HeLa cells transfected with the βWT gene was also assayed to demonstrate that the experimental RPA was carried out in probe excess. (**C**) HeLa cells were transfected with the α-globin constructs (also containing the puro^R^ gene) specified above each lane. Total RNA from transiently transfected HeLa cells was isolated and analyzed by RPA as above in (B). The percentage mRNA values were plotted for each construct, and standard deviations from four independent experiments are shown. Below the graph, the schema represents the studied human α-globin mRNA. Vertical lines represent translation initiation (AUG) or termination (UAA) codons, which are aligned with the X-axis of the graph that shows the mRNA nonsense-mutated codons.

The boundaries were mapped by assessing expression of corresponding series of α- and β-globin mRNAs (Figure 1). The analysis of the β-globin mRNAs (summarized in Figure 1B) revealed that β15 and β23 mRNAs accumulate at normal levels, more specifically at 105% and 93% of the βWT mRNA, respectively (Figure 1B), while levels of β25, β26, and β39 mRNAs were drastically lower, at 21%, 20% and 22% of βWT mRNA, respectively (Figure 1B). The stabilization of β25, β26 and β39 mRNAs in UPF1-depleted cells to WT levels confirmed that the low levels were due to NMD (Supplementary Figure S1A and B). Targeted comparison of β15 and β39 mRNAs further confirmed the distinction of NMD sensitive and NMD resistant mRNAs related to their AUG-proximity (Supplementary Figure S1C). Together, these data map the boundary of the AUG-proximity effect for NMD resistance of β-globin mRNA between codons 23 and 25.

In a separate experiment, we mapped the boundary of the AUG-proximity effect for NMD resistance in the hα-globin mRNA. Results revealed that α-globin mRNAs carrying a PTC located at exon 1 (exon 1 encompasses 30 codons) accumulate to steady-state levels of 57% to 95% of normal control that are significantly above typical NMD levels (35) (Figure 1C). In contrast, mRNAs containing nonsense mutations in the first half of exon 2, between codons 32 and 70, appear to be fully sensitive to NMD with levels ranging from 27% to 40% of the αWT levels (Figure 1C). Nonsense mutations positioned further into exon 2 achieved higher levels, most likely reflecting an inhibition of NMD as the PTC comes within 50–55 nts of the last exon–exon junction (Figure 1C). As predicted by the EJC model of NMD, the PTCs located 3′ to codon 82 accumulated to normal levels (Figure 1C).

To confirm that the steady-state mRNA levels being measured reflected the corresponding stabilities of the PTC-containing hα-globin mRNAs, we measured the absolute half-lives of selected mRNAs. The half-life of the α32 mRNA (3.5 h) and α40 mRNA (2.0 h) (46) were consistent with a full sensitivity to NMD (Supplementary Figure S2A) and contrasted with the half-lives of α27 (7.5 h) and α30 (7.0 h). The intermediate half-lives of α73 (5.5 h) and α80 (6.2 h) mRNAs are likely to reflect stabilization as the PTC is moved closer to the terminal EJC (47,48).

To directly demonstrate that α-globin transcripts carrying a PTC in exon 1 escape NMD, we analyzed the impact of UPF1-depletion in HeLa cells (Supplementary Figure S2B). In cells treated with control Luc siRNAs, the α7, α10, α12, α14, and α16 mRNAs accumulated to levels 86% to 98% of the αWT control, contrasting with 30% for the NMD-sensitive α40 mRNA. UPF1 depletion had no appreciable impact on the levels of the α7, α10, α12, α14, and α16 mRNAs (Supplementary Figure S2C). Levels of α23, α27 and α30 mRNAs in the control siRNA-treated cells were intermediate (77%, 67% and 66%) (Supplementary Figure S2C), consistent with partial NMD sensitivity. Consistent with this conclusion was the observation that UPF1 depletion resulted in a significant increase in the accumulation levels of α23, α27, α30, and α40 mRNAs (Supplementary Figure S2C), as well as in a significant increase in the half-lives of α27, and α40 mRNAs, while that of the α16 mRNA did not significantly change (Supplementary Figure S2D).

Taken together, these data are consistent with NMD-resistance of PTCs located in close proximity to the AUG. In the case of the hα-globin mRNA, PTCs extending to at least codon 16 escape the full impact of NMD and PTCs located more distal to the AUG in exon 1 demonstrate partial commitment to NMD. Of note, the boundary for the AUG-proximity effect appears to differ between the two human globin mRNAs; the boundary of the AUG-proximity effect for NMD resistance in hβ-globin mRNA maps between codons 23 and 25, contrasting with the boundary further 3′ (between codons 30 and 32) in the hα-globin mRNA. Defining the basis for this difference has potential to the full understanding of the NMD pathway response.

### The human α-globin mRNAs can activate efficient translational re-initiation 3′ to an AUG-proximal PTC

**Figure 2.**
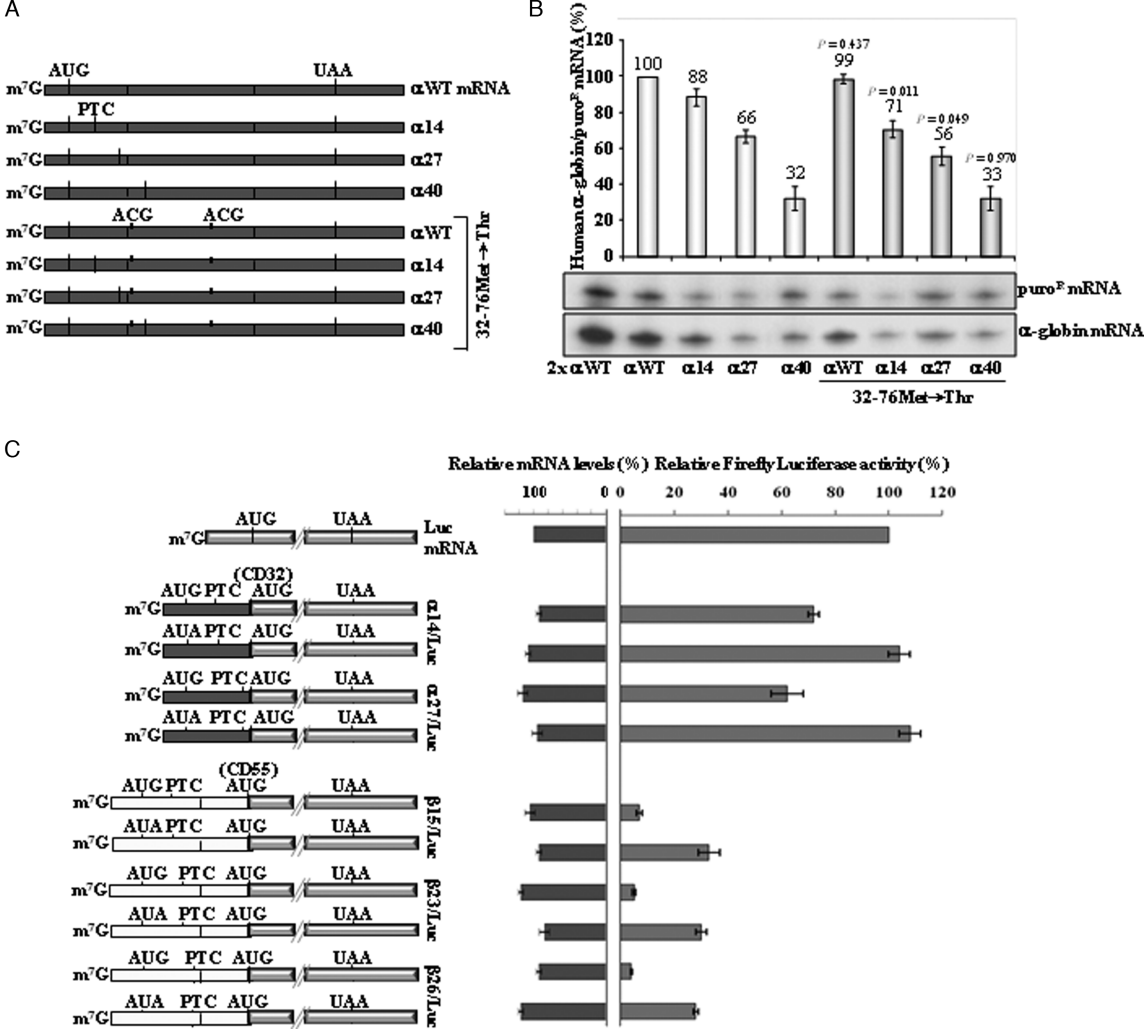
Contrary to what occurs in β-globin mRNA, α-globin mRNAs allow for efficient translation re-initiation, but only partially contributes to NMD inhibition. (**A**) Schematic representation of the studied human α-globin mRNAs. Vertical lines represent translation initiation (AUG) or termination [native (UAA) or premature (PTC)] codons. The conversion of the two potential re-initiation AUGs, at codons 32 and 76, to ACG codons is indicated by short and thick vertical lines. The name of each transcript is specified on the right. (**B**) A representative RPA of RNA isolated from HeLa cells transiently transfected with each gene construct is shown. Identification of the α-globin and the puromycin-resistance (puro^R^) protected fragments is indicated to the right of the autoradiograph. Levels of α-globin mRNA were quantified relatively to the puro^R^ mRNA, and these values are plotted above each respective lane (average and standard deviations) normalized to the expression level of the αWT gene. A 2-fold RNA sample (2x αWT) from HeLa cells transfected with a αWT gene was also analyzed to demonstrate that the experimental RPA was carried out in probe excess. For each case, three independent experiments were performed. *P*-values were determined using a student's *t*-test and refer to the comparison with the corresponding original nonsense-mutated transcript levels, after normalization to the wild-type control. (**C**) The 5′-part of α-globin gene upstream of codon 32 (AUG), or upstream of codon 55 (AUG) of β-globin gene, was fused with the firefly luciferase open reading frame (ORF). Volume-shaped rectangles represent luciferase ORFs, dark-gray rectangles represent α-globin exons and light-gray rectangles represent β-globin exons. The initiation or potential re-initiation, as well as termination [native (UAA) or premature (PTC)] codons, are represented as black vertical lines. A positive control construct was created for each hybrid gene, in which the translation initiation codon for α- or β-globin gene was mutated to AUA. HeLa cells were transiently cotransfected with firefly and *Renilla* luciferase-containing plasmids. Luciferase activity was analyzed 16 h after transfection. In parallel, both firefly and *Renilla* luciferase mRNA levels were determined by semi-quantitative RT-PCR. The activities of firefly and *Renilla* luciferase were calculated relative to their respective mRNA levels (Relative mRNA Levels, left bars), and then the resulting values for firefly luciferase activity/mRNA were quantified relative to those of *Renilla* luciferase activity/mRNA. The obtained luciferase relative activities from hybrid mRNAs were normalized to the relative activity of the uncoupled firefly luciferase (Luc). The average values and standard deviations from three independent experiments corresponding to three independent sets of transfections are shown.

Translation re-initiation downstream of a PTC has the potential to inhibit NMD *via* disruption of downstream EJC(s) (49). We have previously shown that translation re-initiation does not occur 3′ to AUG-proximal nonsense mutations in hβ-globin mRNAs (for example, β15 mRNA) (41). In contrast, re-initiation can occur in the hα-globin mRNA (46). Such re-initiation might account for the difference in the NMD resistance boundary. To test this possibility, we first focused our studies on the α-globin mRNA with a PTC at the 3′ part of exon 1, at codon 27. The PTC at this position in hα-globin mRNA is partially NMD-resistant and is 3′ to the AUG-proximity boundary determined for the hβ-globin mRNA. We mutated the putative initiating AUG codons located downstream of the PTC, which are located at positions 32 and 76. The 32Met and 76Met AUG-to-ACG conversions created the α27.32–76Met→Thr gene (Figure 2A). Its expression was compared to that of the αWT.32–76Met→Thr, α14.32–76Met→Thr, and α40.32–76Met→Thr control genes previously described (46). The α40.32–76Met→Thr mRNA was expressed at the same level as α40 mRNA (33% of αWT), consistent with no translation re-initiation at codon 76 and full NMD commitment (Figure 2B). On the other hand, α14.32–76Met→Thr transcripts accumulate at about 71% of αWT, and at about 80% of α14 mRNA, as previously described (46), showing that blocking potential translation re-initiation sites results in partial NMD triggering of the α14 mRNA. Consistent with this conclusion is the observation that UPF1 depletion resulted in a significant increase in the accumulation of α14.32–76Met→Thr mRNA (Supplementary Figure S3B). In addition, we observed that α27.32–76Met→Thr mRNA accumulates at about 56% of αWT, and at about 85% of α27 mRNA (Figure 2B). Of note, expression of the α27 mRNAs with the double missense mutation remains well above the levels of the α40 mRNA, indicating that translation re-initiation does not fully explain their NMD resistance.

To better quantify the impact of translation re-initiation on α14 and α27 mRNA accumulation, we used a luciferase reporter assay. The first in site for potential re-initiation downstream of α14 and α27 is at codon 32Met. Thus, we created a set of α14/Luc and α27/Luc constructs in which the α-globin gene upstream of codon 32 was fused with the firefly luciferase ORF (Figure 2C). After transient expression of these constructs in HeLa cells, luciferase activities were measured and normalized to the corresponding mRNA levels. Results show that the α14/Luc and α27/Luc constructs carrying the 0Met codon mutated to AUA, allow for 104% and 108% of the uncoupled luciferase relative activity, respectively. In addition, both α14/Luc and α27/Luc constructs lead to the production of considerable high amounts of luciferase, being about 75% and 62% of the uncoupled luciferase activity, respectively (Figure 2C). Taken together, these results demonstrate that translation can efficiently re-initiate at codon 32Met in hα-globin mRNA. Our data further reveal that the efficiency of translation re-initiation decreases when the ORF is lengthened and the intercistronic region is shortened, as has been previously described (43,50).

An equivalent analysis was performed to show the ineffectiveness of β-globin mRNA to re-initiate translation at codon 55Met as previously defined (41). Indeed, we observed that β15/Luc and β23/Luc constructs result in very low amounts of luciferase activity, at about 7% and 5%, respectively, when compared with the relative activity resulting from the uncoupled luciferase gene (Figure 2C). Also, these levels are similar to those obtained from the β26/Luc construct, which shows a luciferase activity of 4% (Figure 2C). The very low luciferase activity given by β26/Luc construct is not surprising, as β26 transcripts are efficiently degraded by NMD. Thus, NMD-resistant (β15 and β23 mRNAs) and NMD-sensitive transcripts (β26) show similar low levels of translation re-initiation.

This set of experiments lead us to conclude that the level of mRNA accumulation for hα-globin mRNAs with AUG-proximal PTCs, and the corresponding resistance to NMD, is impacted to some extent by the potential to re-initiate translation 3′ to the AUG-proximal PTC.

### Human α-globin mRNAs carrying an AUG-proximal PTC do not allow for ribosomal read-through of the PTC

**Figure 3.**
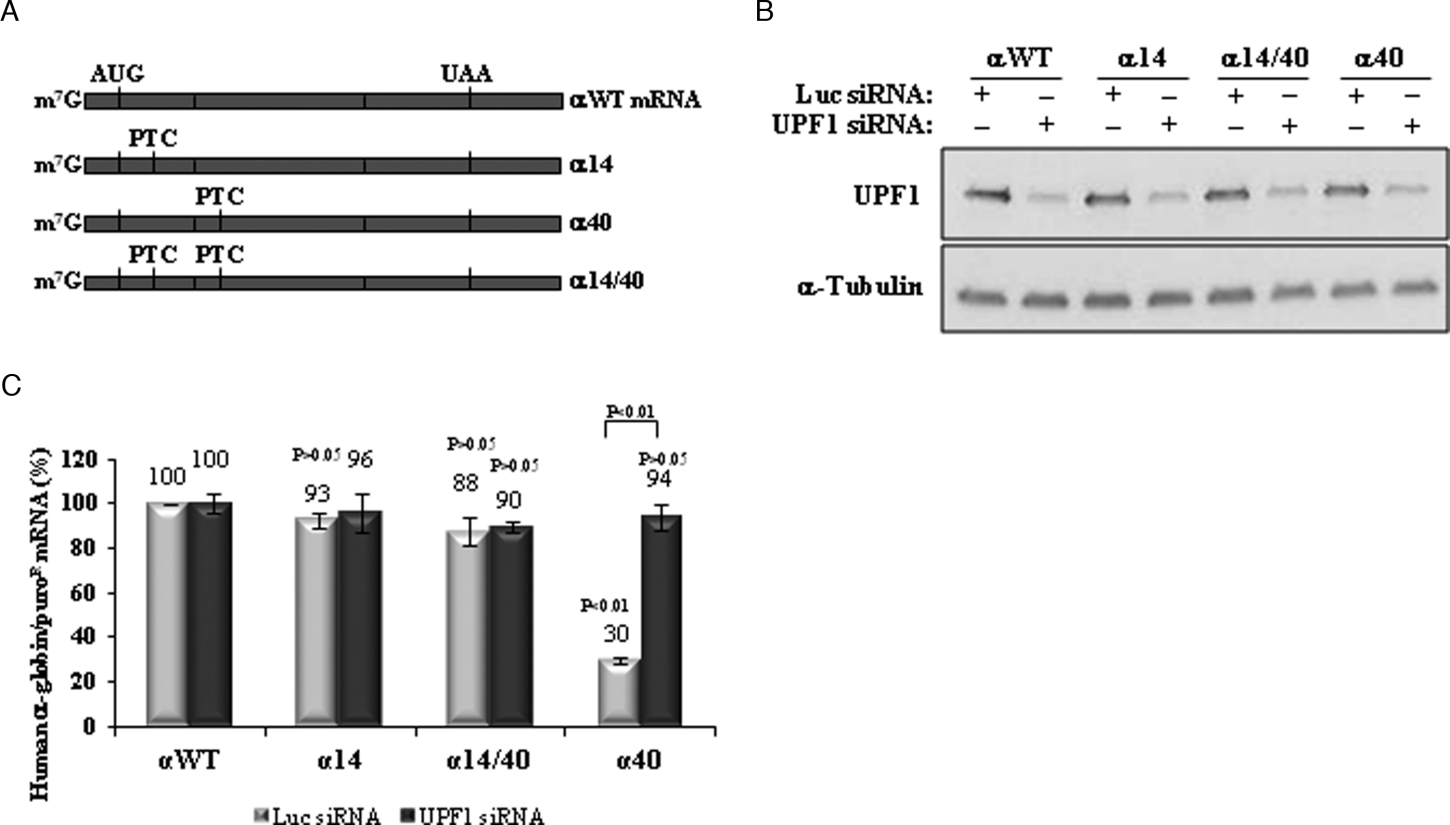
The α-globin mRNA does not allow PTC read-through. (**A**) Schematic representation of the studied human α-globin mRNAs. Vertical lines represent translation initiation (AUG) or termination [native (UAA) or premature (PTC)] codons. The name of each transcript is specified on the right. (**B**) Western blotting analysis of protein samples obtained from HeLa cells transiently transfected with constructs carrying the wild-type α-globin gene (αWT), or a α-globin gene nonsense-mutated at codons 14 (α14), 40 (α40) or 14 and 40 (α14/40). Cells were subjected to single knockdown of UPF1 (UPF1 siRNA) or treated with control siRNA targeting firefly Luciferase (Luc siRNA). Anti-UPF1 and anti-α-tubulin (control) antibodies were used as indicated. (**C**) Using these cells, mRNA levels were determined by RT-qPCR using primers specific for human β-globin gene and for puromicin-resistance (puro^R^) gene. Quantification was performed by the relative standard curve method. Histogram represent fold-change of each sample relative to the control (βWT Luc siRNA) arbitrarily set to 100%. All values are normalized internally to puro^R^ mRNA levels [SD are shown (*n* = 3)]. The *P*-values from student's *t*-tests are also shown. Except otherwise indicated, *P*-values refer to the comparison with the wild-type control transcript levels treated with control siRNA (αWT Luc siRNA).

Ribosomal read-through of a nonsense codon can in theory inhibit NMD, by displacing the downstream located EJC(s) (49) and consequently preventing the interaction of UPF1 with the terminating complex and with the UPF2/UPF3 components of the EJC (7). We have previously shown that the hβ-globin mRNA carrying the β15 mutation in *cis* to the β39 mutation does not allow ribosomal read-through of the β15 PTC (41). These results have been subsequently confirmed by others (51). Here, we assessed the potential contribution of PTC read-through to NMD resistance of AUG-proximal PTCs in the hα-globin mRNA. We constructed an expression vector containing two PTCs *in cis*, one at codon 14 and another at codon 40 (construct α14/40; Figure 3A). The encoded mRNA was expressed in HeLa cells, in parallel with the αWT, α14 and α40 control constructs (Figure 3B). Read-through of the PTC at position 14 would be expected to destabilize the double mutant α14/40 mRNA because the PTC at position 40 is NMD sensitive (Figures 1 and 3C). Contrary to this prediction, we observed that the α14/40 mRNA is expressed at levels comparable to those of the α14 and αWT mRNAs, while α40 mRNA is expressed at 30% of the normal control (Figure 3C). UPF1 depletion of the transfected cells normalized the levels of α40 mRNA as expected, but had no impact on the accumulation of the α14/40 or α14 mRNA. This set of data shows that PTC read-through does not occur and thus cannot account for the NMD resistance of the AUG-proximal nonsense-mutated α-globin mRNAs.

### The boundary of the AUG-proximity effect for NMD resistance is impacted by the ORF sequence

**Figure 4.**
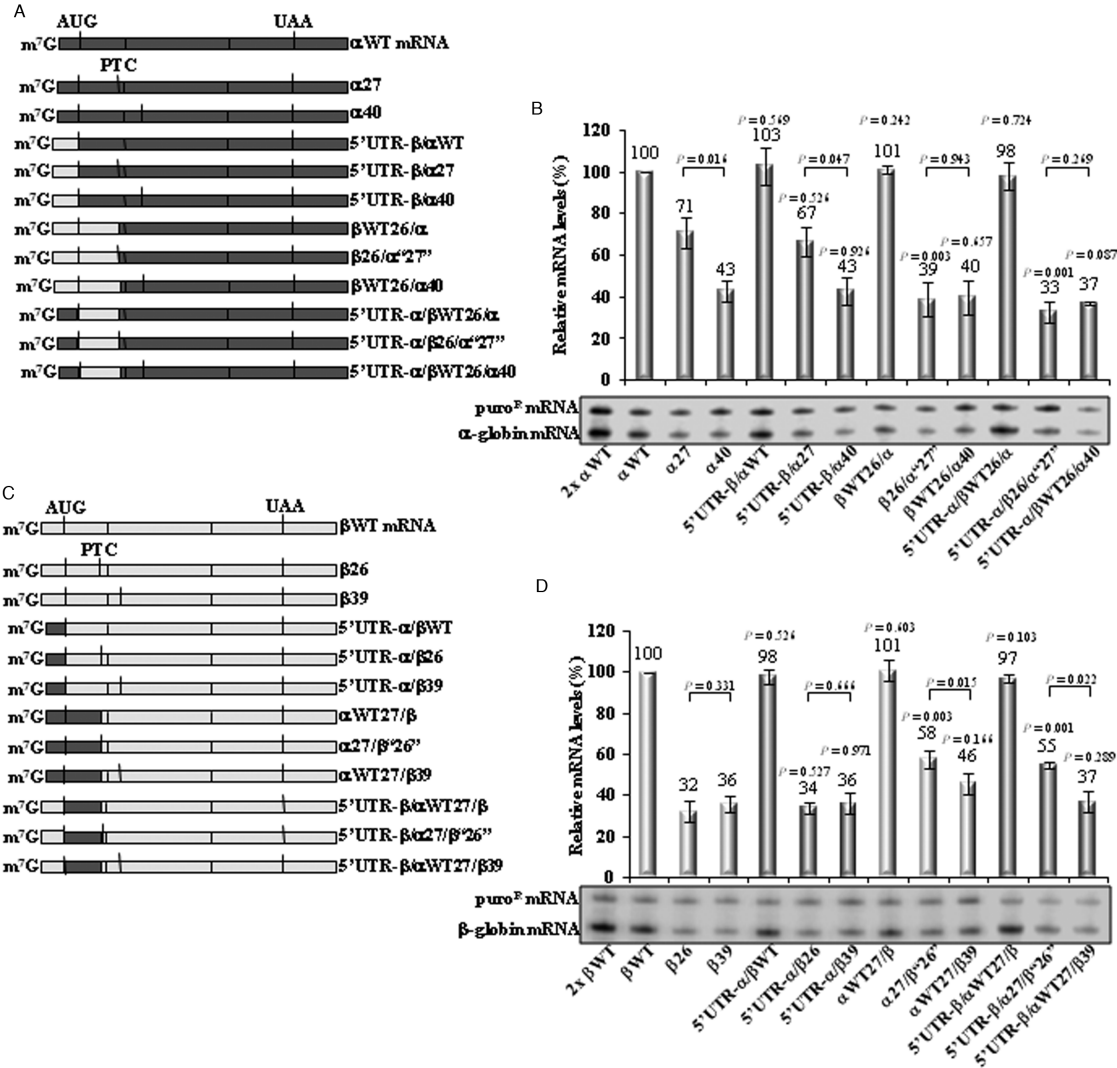
The sequence of the ORF determines the extension of the AUG-proximity effect. (**A**) Physical maps of the α-globin and the hybrid β/α- and α/β/α-globin mRNAs studied. Dark-gray rectangles represent α-globin exons and light-gray rectangles represent β-globin sequences. Translation initiation codons (AUG) and native termination codons (UAA) or premature translation termination codons (PTCs) are depicted by heavy black vertical lines. The name of each mRNA is indicated to its right. (**B**) Representative RPA using RNA isolated from HeLa cells transiently transfected with the constructs specified beneath each lane. The identification of α-globin and the puromycin-resistance (puro^R^) protected fragments is indicated to the left of the autoradiograph. Levels of α-globin mRNA were quantified relatively to the puro^R^ mRNA, and these values are plotted above each respective lane (average and standard deviations from three independent experiments). mRNA levels are normalized to the expression level of the wild-type mRNA (αWT). A 2-fold RNA sample (2x αWT) from HeLa cells transfected with a αWT gene was also analyzed to demonstrate that the experimental RPA was carried out in probe excess. *P*-values were estimated using a student's *t*-test. Except otherwise indicated, *P*-values refer to the comparison with the corresponding original nonsense-mutated transcript levels, after normalization to the wild-type control. (**C**) Schematic representation of the studied β-globin and hybrid α/β- and β/α/β-globin mRNAs, using the same depiction code as for (A) (see above). (**D**) Illustrative RPA of RNA from HeLa cells transiently transfected with the constructs identified beneath each lane. Identification of the β-globin and puro^R^ protected fragments is indicated to the left of the autoradiograph. Average levels (and standard deviations from three independent experiments) of β-globin mRNA (plotted above each respective lane) were measured relatively to the puro^R^ mRNA as in (B) (see above). Except otherwise indicated, *P*-values refer to the comparison with the corresponding original nonsense-mutated transcript levels, after normalization to the wild-type control.

Although human α- and β-globin genes derive from the same ancestral gene and share a similar structure and function, the respective mRNAs are distinct in sequence, with a strong divergence in the structures of the corresponding 5′ UTRs. This difference in sequence is likely to contribute to the lower efficiency for initiating translation in α-globin mRNA (52). Knowing that NMD mechanisms are translation-dependent, we hypothesized that the difference in the boundary of the AUG-proximity effect for α- and β-globin mRNAs might reflect their respective translation initiation efficiencies. This hypothesis was tested by replacing the 5′ UTR of α-globin with that of the β-globin mRNA in αWT, α27 and α40 genes, generating three hybrid mRNAs: 5′ UTR-β/αWT, 5′ UTR-β/α27 and 5′ UTR-β/α40 (Figure 4A). mRNA quantification of these transcripts transiently expressed in HeLa cells showed that the partial NMD inhibition observed for α27 mRNA—it accumulates at 71% of αWT mRNA (Figure 4B)—is maintained in α27 mRNAs with the β-globin 5′ UTR (5′ UTR-β/α27 mRNA is 67% of the αWT).

Next, we tested whether the ORF sequence affects the boundary of the AUG-proximity effect. Thus, in αWT, the entire sequence upstream of codon 27 was replaced by the corresponding sequence of βWT or β26, creating the βWT26/α and β26/α‘27’ constructs, respectively. In α40 gene, the entire sequence upstream of codon 27 was replaced by the corresponding sequence of βWT, generating the βWT26/α40 gene (Figure 4A). Moreover, these latter hybrid genes were further altered by replacing the 5′ UTR of β-globin for that of α-globin, originating the 5′ UTR-α/βWT26/α, 5′ UTR-α/β26/α‘27’ and 5′ UTR-α/βWT26/α40 constructs (Figure 4A). Our results show that α27 mRNAs become fully committed to NMD if they carry the β-globin ORF sequence, regardless of the 5′ UTR sequence (Figure 4B). As a control for NMD, we observed that hybrid transcripts nonsense-mutated at position 40 (5′ UTR-β/α40, βWT26/α40 and 5′ UTR-α/βWT26/α40) accumulate at levels equivalent to those of α40 mRNA (43%, 43%, 40% and 37% of the αWT mRNA, respectively), all being fully committed to decay.

The reciprocal approach was also used. The 5′ UTR of β-globin was replaced by the α-globin 5′ UTR in the βWT, β26 and β39 genes, creating the 5′ UTR-α/βWT, 5′ UTR-α/β26 and the 5′ UTR-α/β39 constructs, respectively (Figure 4C). Analysis of these constructs revealed that the β26 and β39 low mRNA levels are preserved if the transcripts carry the α-globin 5′ UTR (Figure 4D).

Moreover, in the βWT gene, the entire sequence upstream of codon 27 was replaced by the corresponding sequence of αWT or α27 genes, creating the αWT27/β and the α27/β‘26’ genes, respectively; in the β39 gene, the entire sequence upstream of the codon 27 was replaced by the corresponding sequence of αWT, originating the αWT27/β39 gene (Figure 4C). Additionally, the 5′ UTR of α-globin was replaced by the β-globin 5′ UTR in the latter three hybrid genes, which originated the 5′ UTR-β/αWT27/β, 5′ UTR-β/α27/β‘26’, and 5′ UTR-β/αWT27/β39 constructs (Figure 4C). Results show that if β26 mRNA carries the α-globin ORF, irrespectively of the 5′ UTR, the mRNA levels significantly increase from 32% to 58% (α27/β‘26’ mRNA) or 55% (5′ UTR-β/α27/β‘26’ mRNA), relatively to the βWT mRNA level (Figure 4D).

The analysis of these hybrid mRNAs leads us to make two conclusions. First, we find that the sequence of the 5′ UTR has no impact on determining the NMD sensitivity of the transcript. Second, these results reveal an important role of the ORF sequence in determining the full NMD commitment, and thus in determining the exact boundary of the AUG-proximity effect.

### The mRNA secondary structure dictates the boundary of the AUG-proximity effect

**Figure 5.**
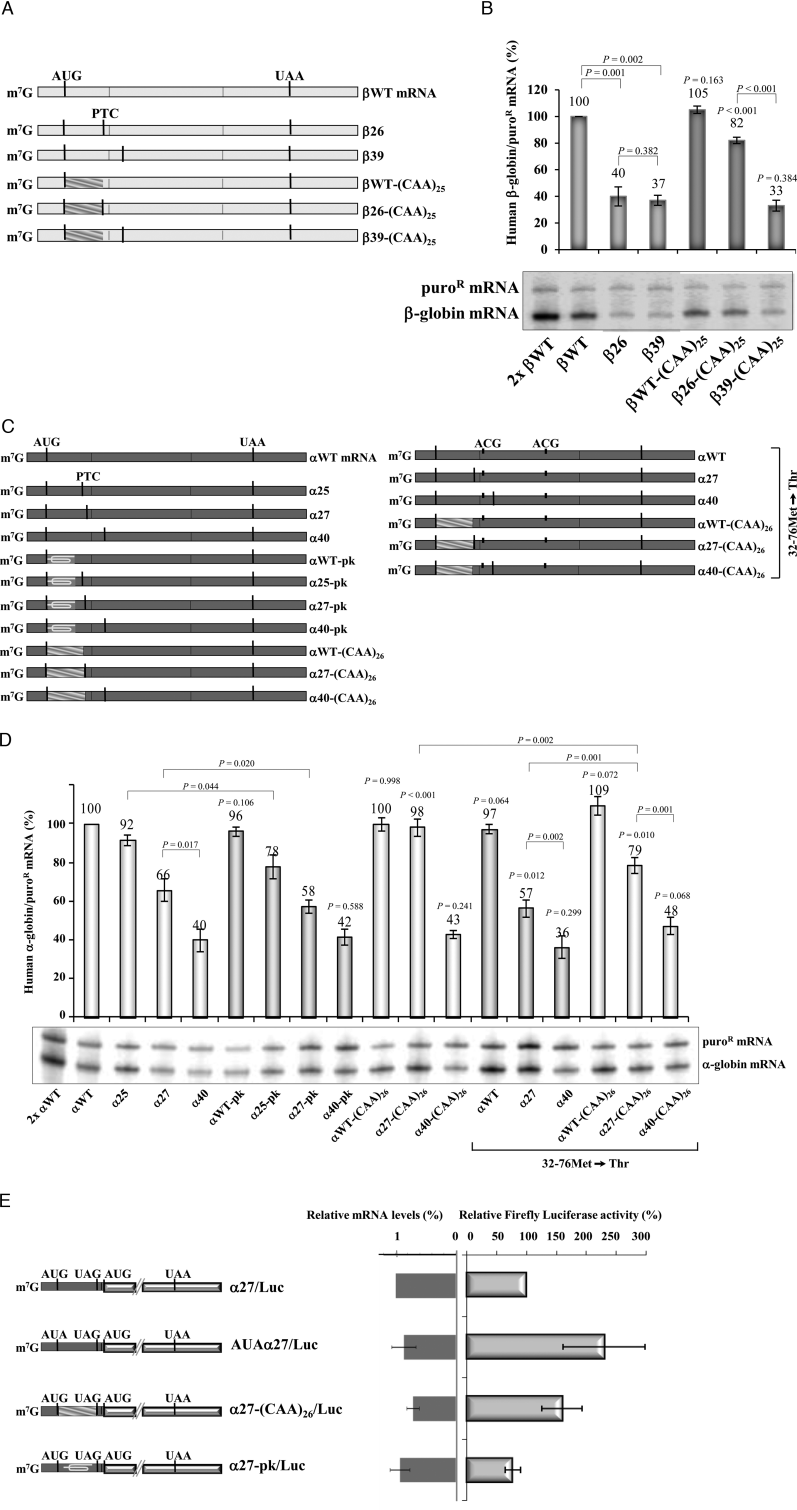
The boundary of the AUG-proximity effect for NMD resistance is determined by the mRNA secondary structure. (**A**) Schematic representation of the studied human β-globin mRNAs. Heavy black vertical lines indicate the positions of translation initiation codons (AUG) and natural (UAA) or premature termination codons (PTCs). Diagonally-striped rectangles represent 25 consecutive ‘CAA’ triplets [(CAA)_25_]. The identification of each transcript is indicated to the right. (**B**) Representative RPA of RNA isolated from HeLa cells transiently transfected with the constructs specified beneath each lane. The identification of β-globin and the puromycin-resistance (puro^R^) protected fragments is indicated to the left of the autoradiograph. Levels of β-globin mRNA were quantified relatively to the puro^R^ mRNA, and these values are plotted above each respective lane (average and standard deviations from three independent experiments) normalized to the expression level of βWT mRNA. A 2-fold RNA sample (2x βWT) from HeLa cells transfected with the βWT gene was also analyzed to ascertain that the RPA was carried out in probe excess. *P*-values were determined using a student's *t*-test. Except otherwise indicated, *P*-values refer to the comparison with the corresponding original nonsense-mutated transcript levels, after normalization to the wild-type control. (**C**) Schematic representation of the studied human α-globin mRNAs, using the same depiction code as for (A) (see above). Loop shapes in dark rectangles represent pseudoknot (pk) structures. The alteration of the two potential re-initiating AUGs, at codons 32 and 76, to ACG triplets is indicated by short and thick vertical lines (32–76Met→Thr). The identification of each mRNA is indicated to the right of the diagram. (**D**) Representative RPA using RNA isolated from HeLa cells transiently transfected with the constructs specified beneath each lane. The positions of α-globin and the puromycin-resistance (puro^R^) protected fragments are indicated to the right of the autoradiograph. Resulting levels of α-globin mRNA were quantified as in (B). Except otherwise indicated, *P*-values refer to the comparison with the corresponding original nonsense-mutated transcript levels, after normalization to the wild-type control. (**E**) Luminometry assay to test translation re-initiation after translation of a highly structured (pseudoknot; pk) or unstructured [(CAA)_26_] ORF. The 5′-part of α-globin gene upstream of codon 32 (AUG), was fused with the firefly luciferase ORF at its translation initiation codon. Volume-shaped rectangles represent luciferase exons, dark-gray rectangles represent α-globin sequences. The initiation or potential re-initiation, as well as termination [native (UAA) or premature (UAG)] codons, are represented as black vertical lines. A control construct was created, in which the α-globin translation initiation codon was mutated to AUA allowing to control for the maximum of luciferase activity. HeLa cells were transiently cotransfected with firefly and *Renilla* luciferase-containing plasmids and protein levels were analyzed as above for Figure 2C.

The rate of translation elongation is sensitive to secondary and higher order structures of the translated ORF (53). Structural constraints of mRNA, such as stem-loops or pseudoknots, are known to retard the progression of elongating ribosomes (54). We have previously demonstrated that insertion of a pseudoknot (43) into the ORF of β-globin mRNA containing an AUG-proximal PTC, converts it from NMD resistant to NMD sensitive (21). This response and data from Figure 4, suggested a model in which the time taken by the ribosome to translate the ORF dictates the boundary of the AUG-proximity effect. To further explore this potential determinant of NMD and the determinants of the boundary of the AUG-proximity effect, we next replaced the first 25 codons of the β26 gene by a tandem array of 25 ‘CAA’ repeats, which has been shown to facilitate rapid translation elongation (55), creating the β26-(CAA)_25_ construct (Figure 5A). Knowing that the boundary of the AUG-proximity effect in the hβ-globin mRNA is located between codons 23 and 25, the unstructured sequence when inserted upstream of the β26 PTC should inhibit NMD and shift the boundary of the AUG-proximity effect further downstream. The first 25 codons of the βWT, and β39 control genes were also replaced, creating the βWT-(CAA)_25_, and β39-(CAA)_25_ constructs, respectively (Figure 5A). mRNA secondary structures predicted by MFOLD (http://mfold.bioinfo.rpi.edu/cgi-bin/rna-form1.cgi) show that while the β26 mRNA has a minimal free energy of −232.50 kcal/mol, introduction of the (CAA)_25_ unstructured segment makes this mRNA to increase its minimal free energy to −199.90 kcal/mol. Our analysis of mRNA accumulation shows that the altered β26 mRNA [β26-(CAA)_25_] increased from 40% to 82% of the wild-type control (Figure 5B). In contrast, the unstructured insertion did not relieve NMD in the β39 mRNA: β39, and β39-(CAA)_25_ mRNAs accumulate at similar levels, respectively at 37%, and 33% of the βWT transcript (Figure 5B). As expected, expression of both β39 mRNAs was brought to WT levels in UPF1-depleted cells (Supplementary Figure S4).

To further relate ORF structure to AUG-proximity boundary, using the α-globin mRNAs, we introduced either a pseudoknot-inducing sequence or an unstructured CAA repeat in the ORF. The pseudoknot was introduced into αWT, α25, α27 and α40 genes, replacing the first 19 codons of these constructs (Figure 5C). MFOLD predicted that the pseudoknot induces a significant change in the minimal free energy from −231.60 kcal/mol (α25) and −225.10 kcal/mol (α27) to −241.20 kcal/mol and −228.40 kcal/mol, respectively. In parallel, the unstructured (CAA)_26_ repeats were introduced into the αWT, α27 and α40 mRNAs (see Figure 5C) changing the minimal free energy of the hα-globin mRNAs from −225.10 kcal/mol to −185.70 kcal/mol. The analyses of mRNA accumulation revealed that the pseudoknot structure introduced into the α25 gene (α25-pk gene) resulted in a significant reduction of the encoded mRNA accumulation levels (Figure 5D). Identical outcomes were obtained when the pseudoknot structure was inserted *in cis* to the PTC at position 27 (Figure 5D). In contrast, the unstructured ORF consisting in 26 ‘CAA’ repeats inserted into the α27 mRNA [α27-(CAA)_26_ mRNA] significantly increased the mRNA levels from 66% to 98%, relatively to the αWT (Figure 5D). As expected, in the context of a transcript with a PTC further downstream already NMD-sensitive (α40), the pseudoknot structure, or the (CAA)_26_ unstructured ORF, does not affect levels of mRNA accumulation (Figure 5D). These results lead us to conclude that the sensitivity of mRNAs with AUG-proximal PTCs to NMD is impacted by the strength of secondary structure preceding the PTC; the presence of a pseudoknot increases NMD sensitivity and the presence of an unstructured ORF decreases NMD sensitivity.

Knowing that hα-globin transcripts carrying an AUG-proximal PTC, such as the α27 mRNA, allow for efficient downstream translation re-initiation, which is partially responsible for their NMD inhibition (Figure 2), we next investigated the outcome of the (CAA)_26_ unstructured ORF independently of the translation re-initiation effect. For that, we introduced the 26 consecutive ‘CAA’ repeats into the αWT.32–76Met→Thr, α27.32–76Met→Thr and α40.32–76Met→Thr genes. Under these conditions, the presence of the (CAA)_26_ segment into the ORF of the α27 mRNA makes it to increase from 57% to 79% of the αWT mRNA level (Figure 5D). These results are parallel to those obtained when translation re-initiation is allowed (Figure 5D). In fact, by comparing results obtained in both conditions, we observe that the presence of the unstructured ORF into the α27 mRNA makes a 1.5-fold increase in mRNA levels. Thus, our data show that in the α27 transcripts, the unstructured ORF enhances NMD inhibition independently of translation re-initiation. However, it is worth noticing that the accumulation levels for α27 and α27-(CAA)_26_ mRNAs are higher when translation re-initiation is allowed (Figure 5D). These results show that under conditions where translation re-initiation can occur, NMD inhibition is stronger as it results from two different effects: translation re-initiation and the AUG-proximity effect. In addition, the AUG-proximity effect for NMD inhibition is more prominent when the ORF has an unstructured sequence.

To confidently ascribe the impact of the pseudoknot and CAA repeats to their impact on translational elongation, we next confirmed that in fact the unstructured ORF decreases the time taken by the ribosome to translate the ORF, while the pseudoknot sequence in the ORF increases the time of translation elongation. For that, the α-globin sequence downstream of the codon 32, in α27, α27-(CAA)_26_, and α27-pk gene constructs, was replaced by the firefly luciferase (Luc) ORF, creating α27/Luc, α27-(CAA)_26_/Luc, and α27-pk/Luc genes, respectively (Figure 5E). To normalize results to the effectiveness of the 32Met codon to initiate translation, we used the AUAα27/Luc gene that has the first AUG mutated to AUA (Figure 5E). Luciferase activities were measured and normalized to the mRNA levels of the corresponding construct to obtain translation efficiencies. Results show that the unstructured ORF leads to the production of more luciferase protein than the α27/Luc mRNA, since luciferase activity from α27-(CAA)_26_/Luc mRNA is at 153% of that from α27/Luc (Figure 5E). On the other hand, the α27-pk construct allows for 67% of relative luciferase activity comparing to that from α27/Luc mRNA (Figure 5E). Knowing that efficiency of translation re-initiation is directly proportional to the elongation rate (50), our data show that the (CAA)_26_ sequence in the ORF allows a higher elongation rate than the α27 or pseudoknot sequences. Taken our results together, we can conclude that the ORF secondary structure by affecting the elongation rate, dictates the boundary of the AUG-proximity effect for NMD inhibition.

## DISCUSSION

In general, nonsense codons located more than 50–55 nts upstream of the 3′-most exon–exon junction elicit NMD in mammalian cells (47,48). However, our previously reported data have shown that human β-globin transcripts carrying nonsense mutations in the 5′ region of exon 1 accumulate to levels comparable to those of wild-type β-globin mRNA (40). This ability of mutated β-globin mRNA to escape NMD was demonstrated to depend on the distance of the nonsense codon to the initiator AUG (21,41,46) and, accordingly, it was named the ‘AUG-proximity effect’. Additional data have supported that this effect is due to the influence of PABPC1, which seems to be brought into the proximity of an early PTC during cap-dependent translation and 43S scanning (21,34). Although this effect has not been observed in budding and fission yeast (56,57), it seems to be a general attribute in mammalian cells (21); these differences might reflect differences in the NMD determinants that appeared during evolution. For example, contrary to what occurs in mammalian cells, in *S. pombe*, the important NMD determinant is the proximity of the PTC to an intron, being modest the contribution of the distance between the PTC and PABPC1 (57).

The aim of this study was to analyze if human α- and β-globin mRNAs share similar NMD profiles. Being two highly related genes, α- and β-globin preserve a similar general organization, and the encoded peptides form a comparable structure and accomplish an equivalent function. Therefore, it is not surprising that they share the overall NMD behavior, and the AUG-proximity effect for NMD inhibition observed in β-globin (Figure 1B) was also found in α-globin mRNA (Figure 1C). However, we have observed that this effect occurs for PTCs located further downstream (until codon 30) in the α-globin mRNA (Figure 1). On the other hand, contrary to what is observed for β-globin mRNA, the human α-globin mRNA exhibits efficient translation re-initiation at a downstream AUG, for example at codon 32 (Figure 2 and reference 46). Translation re-initiation contributes to NMD inhibition, as observed in Figure 2B. However, the degree of translation re-initiation observed in short ORF-containing α-globin transcripts is insufficient to explain the full outcome of NMD inhibition (Figure 2B). Indeed, our results show that NMD inhibition observed in α-globin transcripts carrying a short ORF is due to both the AUG-proximity effect and translation re-initiation, while for β-globin transcripts carrying an AUG-proximal PTC, NMD inhibition is fully explained by the AUG-proximity effect as translation re-initiation is very modest (Figure 2C). The potential role of translation re-initiation in NMD resistance of AUG-proximal nonsense-mutated β-globin mRNAs has been the subject of a previous study (51). In that study, the NMD-resistance of β-globin AUG-proximal nonsense-mutated mRNAs was attributed to efficient translation re-initiation downstream of the PTC. It may be of importance, however, to note that the interpretation of these studies is complicated by the use of a β-globin gene in which exon 1 was transformed into a functional exon 2 by the introduction of an exogenous intron into the 5′ UTR. The expression of this recombinant β-globin gene, even lacking a PTC, was repressed when compared to the normal β-globin transcript (51). In contrast, by using globin genes with native structures, we have found that even in cases in which we do detect some level of translation re-initiation downstream of the AUG-proximal PTC, site-specific elimination of the re-initiation codons fails to fully restore NMD-sensitivity (46). Thus, while a contribution of translation re-initiation in NMD-evasion cannot be completely ruled out in any particular circumstance, our detailed analyses of the human β-globin gene lead us to conclude that the AUG-proximity is a major inhibitor of the NMD pathway.

Our results show that the boundary of the AUG-proximity effect for NMD inhibition in the human α- and β-globin transcripts is determined by each of the ORF secondary structure (Figure 4) that, in turn, affects the time of translation elongation. In fact, an unstructured ORF speeds up progression of elongating ribosomes and inhibits decay of an otherwise NMD-sensitive nonsense-mutated transcript (Figure 5). On the contrary, a complex secondary structure present in a short ORF retards progression of elongating ribosomes and increases NMD efficiency in an otherwise NMD-insensitive nonsense-mutated transcript (Figure 5). Knowing that the mRNA secondary structure is involved in determining the overall rate of translation, since mRNA unwinding causes prolonged ribosome pausing (53,54), results herein suggest that the rate of translation elongation might be higher in α- than in β-globin transcripts, given that the boundary of the AUG-proximity effect is located further downstream in the α-globin mRNA.

In normal individuals, the average α/β-globin mRNA ratio is about 1.3 (58). Of interest, nearly 70% of α-globin mRNA is mainly found in pre-initiation complexes, whereas about 50% of total β-globin mRNA is associated with actively translating 80S ribosomes (52). These data suggest that α-globin manifests an inefficient or at least delayed translation initiation. Therefore, α-globin mRNA may actually have a higher translation elongation rate than β-globin mRNA to assure a correct balance between the synthesized α- and β-globin chains. On the other hand, the α-globin gene is known to possess a high G+C content and this feature leads to a strong union of the DNA double strand. In fact, the α-globin gene cluster presents in average about 60% G+C content and the β-globin cluster about 40% (59). The average G+C content in the non-repetitive fraction of the human genome is also about 40% (60,61). Moreover, the average G+C content of the α-globin cDNA has been determined to be around 62% and that of β-globin cDNA around 51% (62). Thus, it would be expected that the α-globin mRNA could present a secondary structure more robust than that of β-globin mRNA. However, this is apparently not the case, since data regarding the optimal mRNA secondary structure and associated minimum free energy prediction, using the MFOLD Web Server software, attribute a more stable secondary structure to the β-globin mRNA (−230.60 kcal/mol for β-globin mRNA versus −224.80 kcal/mol for α-globin mRNA). The more unstable secondary structure of the α-globin mRNA is also in accordance with the possibility of this transcript to present a higher translation elongation rate than that observed for the β-globin mRNA, and this supports our present data.

**Figure 6.**
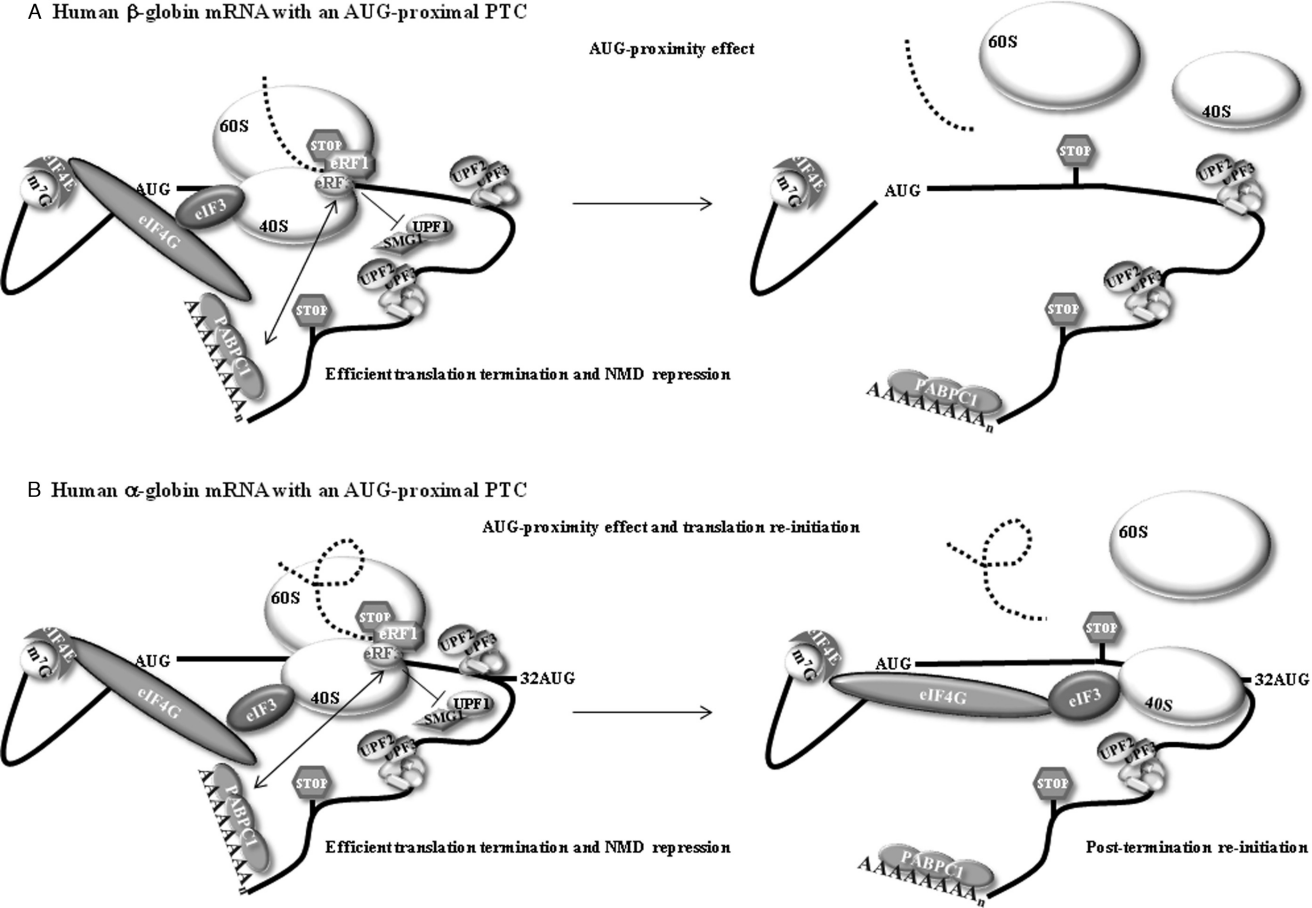
A model for the effect of an AUG-proximal premature termination codon (PTC or stop codon). (**A**) The effect in the human β-globin transcript. During cap-mediated translation initiation, cytoplasmic poly(A) binding protein 1 (PABPC1) interacts with the eukaryotic initiation factor 4G (eIF4G). This interaction indirectly tethers PABPC1 to the 40S ribosomal subunit *via* the interaction of eIF4G with eIF3, which interacts with the 40S. The resulting configuration brings PABPC1 into the vicinity of the AUG initiation codon as a consequence of 43S scanning. The maintenance of the PABPC1-eIF4G-eIF3 interactions with the 40S during the first steps of translation elongation brings PABPC1 into close proximity with the termination complex at an AUG-proximal PTC. This proximity allows PABPC1 to interact with the release factor eRF3, thus impairing the UPF1-eRF3 interaction, resulting in efficient translation termination and inhibition of NMD—this was called the ‘AUG-proximity effect’. (**B**) In the human α-globin transcript, the AUG-proximity effect is observed for PTCs located further downstream because the open reading frame (ORF) is less structured. The more relaxed structure allows a faster elongation rate resulting in a longer window of the AUG proximity effect. In addition, the brief ORF translation also allows efficient translation re-initiation to occur at codon 32. This re-initiation diminishes the levels of residual exon junction complexes on the mRNA, thus contributing to the overall repression of NMD.

The relevance of the time taken to translate a short ORF is related to the fact that some initiation factors appear to remain ribosome-associated for the first moments of the ORF translation elongation (43,50,63,64). Thus, if the elongation phase is brief, the ribosome may reach the translation termination codon before it has had time to discharge initiation factors and other associated proteins. When the ribosome arrives at the stop codon during translation of a short ORF, it might still hold the interaction with eIF4G, due to the brief translation, and PABPC1 might remain bound to eIF4G as well. The proximity of PABPC1 at the end of translation has been shown to promote correct termination and to inhibit NMD activation in human cells (21–24,65) as it competes with UPF1 to interact with eRF3 at the termination complex (7,26,34). The inability of UPF1 to bind eRF3 prevents the interaction between the premature termination complex and UPF2/UPF3 at a downstream EJC, which results in the repression of NMD. The hypothesis of the time taken by the ribosome to translate a short ORF being a major determinant of NMD inhibition can be summarized as follows: during a brief cap-mediated translation of a short ORF, PABPC1 might be in close proximity to the ribosome until it reaches the termination codon. In this situation, PABPC1 may interact with eRF3 and preclude the binding of UPF1 to eRF3, thus preventing NMD activation (Figure 6A). In fact, this model is also in accordance with our previous data (34). This effect will be much more downstream extended if the short ORF is unstructured than if it is a well-structured one. In the unstructured ORF, the ribosome is faster and elongates too further downstream before the initiation factors, such as eIF4G-PABPC1, are disassembled; in addition, by the time the ribosome reaches the PTC, PABPC1 can interact with eRF3 at the terminating complex, which inhibits NMD (Figure 6B). Alternatively, and in accordance with recent data (36,37), PABPC1-bound eIF4G might be the player that represses NMD. Actually, a brief ORF translation, which allows the retention of some initiation factors by the time the ribosome reaches the stop codon, may explain both the AUG-proximity effect (reflecting the proximity of PABPC1 to the PTC) and translation re-initiation, when the transcript is permissive to both mechanisms (Figure 6B). In fact, the resumption of scanning and re-initiation also require a cap-mediated translation initiation with the participation of eIF4F, and most likely depends on the preservation of eIF4F, at least the central one-third fragment of eIF4G, and eIF3 associated with the ribosome throughout translation of the ORF (50,64,66). Our observation that an unstructured ORF in the α-globin mRNAs enhances NMD inhibition due to both the AUG-proximity effect and translation re-initiation further supports this notion. However, for translation re-initiation to occur it is necessary that the intercistronic sequence and the re-initiation codon are favorable (50), and this does not seem to be the case in the β-globin mRNA. In addition, UPF1 seems to play a role in the efficiency with which ribosomes are released from the mRNA after translation termination (39). This effect is likely to modulate the efficiency of translation re-initiation (39). Our results therefore suggest that α- and β-globin mRNAs differently interact with UPF1. The identification of additional features that determine efficient translation re-initiation in α-globin but not in β-globin AUG-proximal nonsense-mutated transcripts will be further studied.

## SUPPLEMENTARY DATA

Supplementary Data are available at NAR Online.

SUPPLEMENTARY DATA
