# Supplementary material for: Resistance of mRNAs with AUG-proximal nonsense mutations to nonsense-mediated decay reflects variables of mRNA structure and translational activity
Source: Nucleic Acids Res. 2015 Jun 11;43(13):6528–44. doi: 10.1093/nar/gkv588 (PMC4513866; doi:10.1093/nar/gkv588)
Supplement: SUPPLEMENTARY DATA [file supp_43_13_6528__index.html]

Resistance of mRNAs with AUG-proximal nonsense mutations to nonsense-mediated decay reflects variables of mRNA structure and translational activity — Resistance of mRNAs with AUG-proximal nonsense mutations to nonsense-mediated decay reflects variables of mRNA structure and translational activity — SUPPLEMENTARY DATA 

# Resistance of mRNAs with AUG-proximal nonsense mutations to nonsense-mediated decay reflects variables of mRNA structure and translational activity

## SUPPLEMENTARY DATA

- SUPPLEMENTARY DATA
