# Supplementary material for: Resistance of mRNAs with AUG-proximal nonsense mutations to nonsense-mediated decay reflects variables of mRNA structure and translational activity
Source: Nucleic Acids Res. 2015 Jun 11;43(13):6528–44. doi: 10.1093/nar/gkv588 (PMC4513866; doi:10.1093/nar/gkv588)
Supplement: SUPPLEMENTARY DATA [file supp_gkv588_nar-00902-r-2015-File009.pdf]

SUPPLEMENTARY DATA

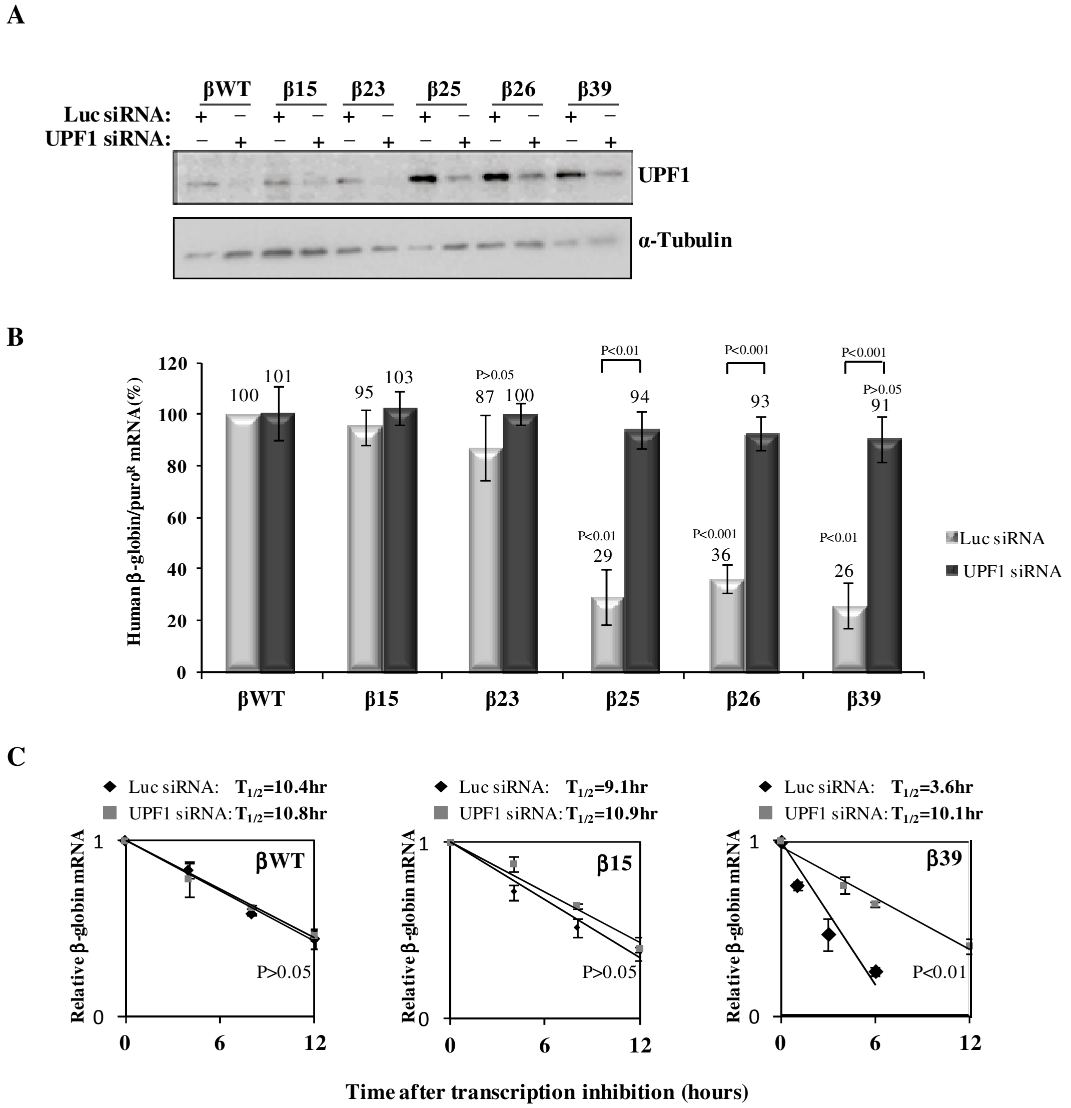

Figure S1. In the β-globin transcript, the boundary of the AUG-proximity for NMD inhibition occurs between codons 23 and 25. (A) Western blotting analysis of protein samples obtained from HeLa cells transiently transfected with wild-type (βWT), NMD resistant (β15, β23) and NMD sensitive (β25, β26, β39) human β-globin constructs. Cells were subjected to single knockdown of UPF1 (UPF1 siRNA) or treated with control siRNA, targeting firefly Luciferase (Luc siRNA). Anti-UPF1 and anti-α-tubulin (control) antibodies were used as indicated. (B) mRNA levels were determined by RT-qPCR using primers specific for human β-globin gene and puro^R cDNAs. Quantification was performed by the relative standard curve method. Histogram represent fold-change of each sample relative to the control (βWT Luc siRNA) arbitrarily set to 100%. All values are normalized internally to puro^R mRNA levels [s.d. are shown (n=3)]. The P-values from student's *t*-tests are also shown. Except otherwise indicated, P-values refer to the comparison with the wild-type control transcript levels treated with control siRNA (βWT Luc siRNA). (C) For mRNAs half-live analyses, total RNA from transfected HeLa cells subjected to knockdown of UPF1 (UPF1 siRNA, grey marker) or treated with control siRNA, targeting firefly Luciferase (Luc siRNA,

black marker) was isolated at various time intervals [βWT, β15: 0, 4, 8, 12 hours; β39: 0, 1, 3, 6 hours] after treatment with 50mg/ml DRB for transcription inhibition and analyzed by RT-qPCR. To calculate the β-globin mRNA half-lives, each data time point was expressed as a ratio of β-globin:puro^R mRNA and normalized to the average value of all time points from a single transfection. The ratios were then renormalized to the average initial time point from all transfections (time 0=1). Each point represents the mean ± standard deviation from three independent experiments. Linear regression analysis was performed by standard techniques. The half-lives ($T_{1/2}$) of the mRNAs and the P-values from student's *t*-tests are indicated.

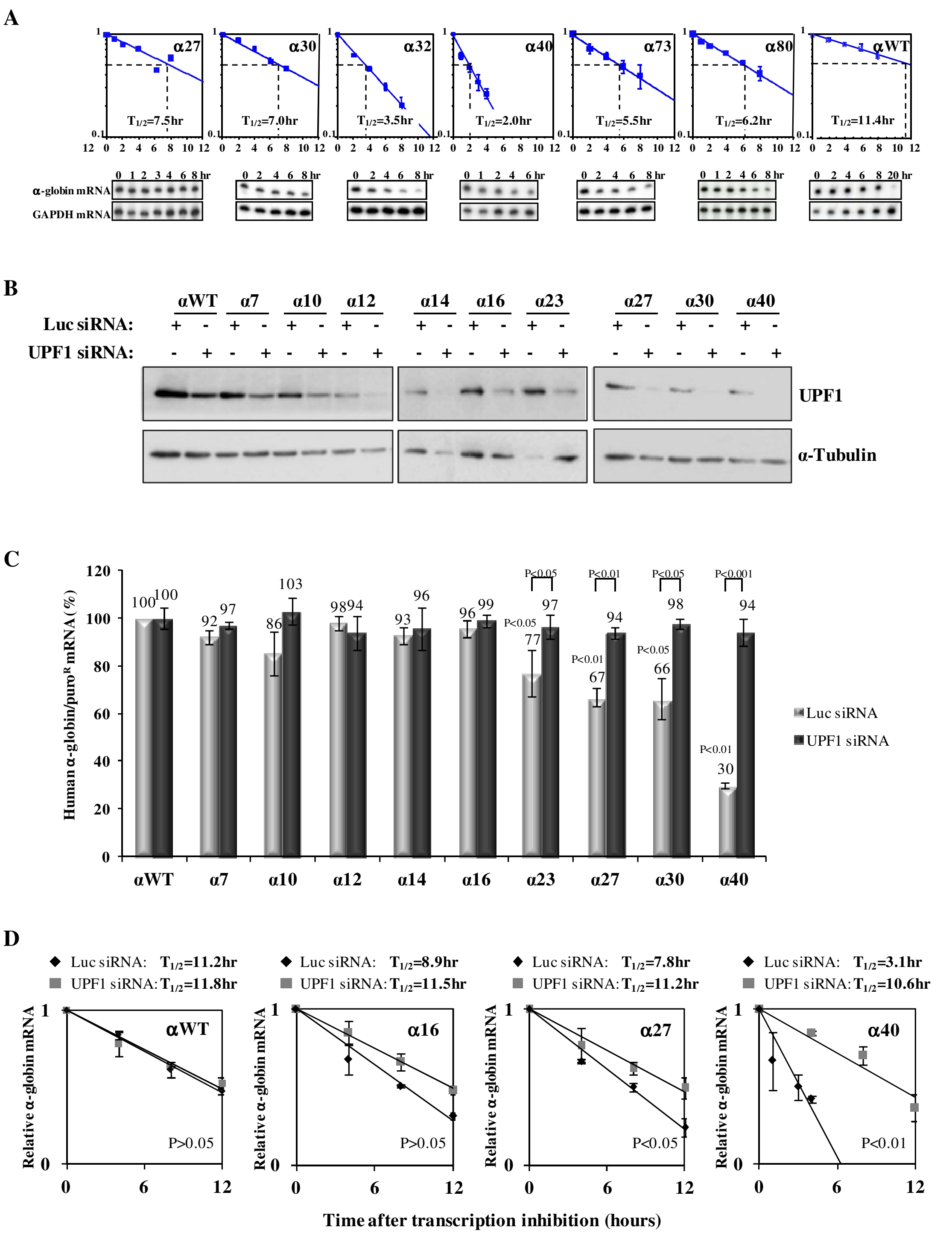

Figure S2. In the α-globin transcript, the boundary of the AUG-proximity for NMD inhibition occurs between codons 30 and 32. (A) Mouse erythroleukemia (MEL) cells stably expressing the tetracycline (tet) transactivator (MEL/tTA cells) were transiently transfected with αWT, α16, α27, α30, α32, α40, α73 or α80 genes under the transcriptional control of a tTA-regulated promoter. Cells were pulsed with the respective α-globin mRNAs for 4 hours (hr) by transfer to tet(-) medium and then transferred back to tet(+) medium for analysis of decay rates. Total mRNA was isolated

at various time intervals during the transcriptional chase period and analyzed by RPA. RNase protection bands corresponding to human α-globin mRNA and to the constitutively expressed mouse GAPDH mRNA (loading control) are indicated. The intensities of the α-globin mRNA bands were quantified and normalized relatively to the GAPDH mRNA band. The data were recorded in the graphs presented. To calculate the α-globin mRNA half-lives, each data time point was expressed as a ratio of α-globin:GAPDH mRNA and normalized to the average value of all time points from a single transfection. The ratios were then renormalized to the average initial time point from all transfections (time 0=1). Each point represents the mean ± standard deviation (s.d) from four independent experiments. Linear regression analysis was performed by standard techniques. The half-lives ($T_{1/2}$) of the mRNAs are indicated. (B) Western blotting analysis of protein samples obtained from HeLa cells transiently transfected with constructs carrying the wild-type human α-globin gene (αWT) or a nonsense-mutated α-globin gene (α7, α10, α12, α14, α16, α23, α27, α30, α40). The cells were subjected to single knockdown of UPF1 (UPF1 siRNA) or treated with control siRNA, targeting firefly Luciferase (Luc siRNA). Anti-UPF1 and anti-α-tubulin (control) antibodies were used as indicated. (C) mRNA levels were determined by RT-qPCR using primers specific for human α-globin and puromicin-resistance ($puro^R$) cDNAs. Quantification was performed as for supplementary figure 1B. (D) For mRNAs half-live analyses, total RNA from transfected HeLa cells subjected to knockdown of UPF1 (UPF1 siRNA, grey marker) or treated with control siRNA targeting firefly Luciferase (Luc siRNA, black marker), was isolated at various time intervals after treatment with 50mg/ml DRB and analyzed by RT-qPCR. Analyses of the α-globin mRNA half-lives were performed as in supplementary figure 1C.

A

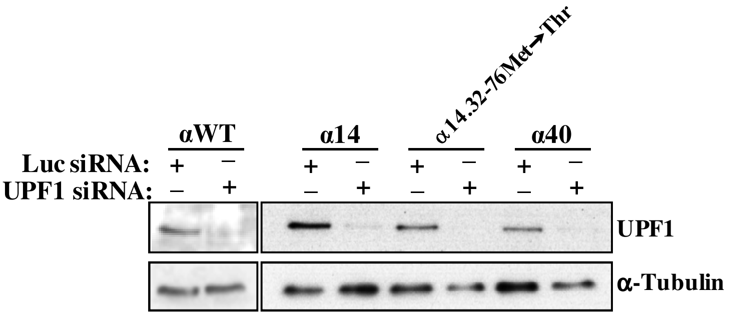

B

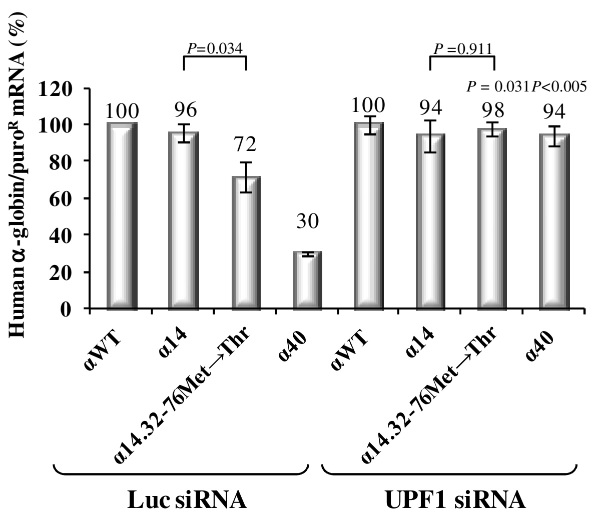

Figure S3. When translation re-initiation is blocked, the lower levels of AUG-proximal α-blogin transcripts are due to the activation of the UPF1-dependent decay pathway. (A) Western blot analysis of HeLa cells extracts transfected with short interfering RNAs for human UPF1 (UPF1 siRNA) or for a nonspecific target, luciferase (Luc siRNA). Twenty-four hours after siRNA treatment, cells were cotransfected with the α-globin constructs specified above each lane, along with a second dose of the corresponding siRNA (UPF1 or Luc). Twenty-four hours after constructs transfection, protein and RNA were isolated from the cells. Immunoblotting was performed using a human UPF1 specific antibody and a α-tubulin specific antibody to control for variations in protein loading. (B) Depletion of UPF1 increases α14.32-76Met→Thr mRNA to normal levels. mRNA levels were determined by RT-qPCR using primers specific for human β-globin gene and for the puromicin-resistance (puro^R) gene. Quantification was performed as for supplementary figure S1B.

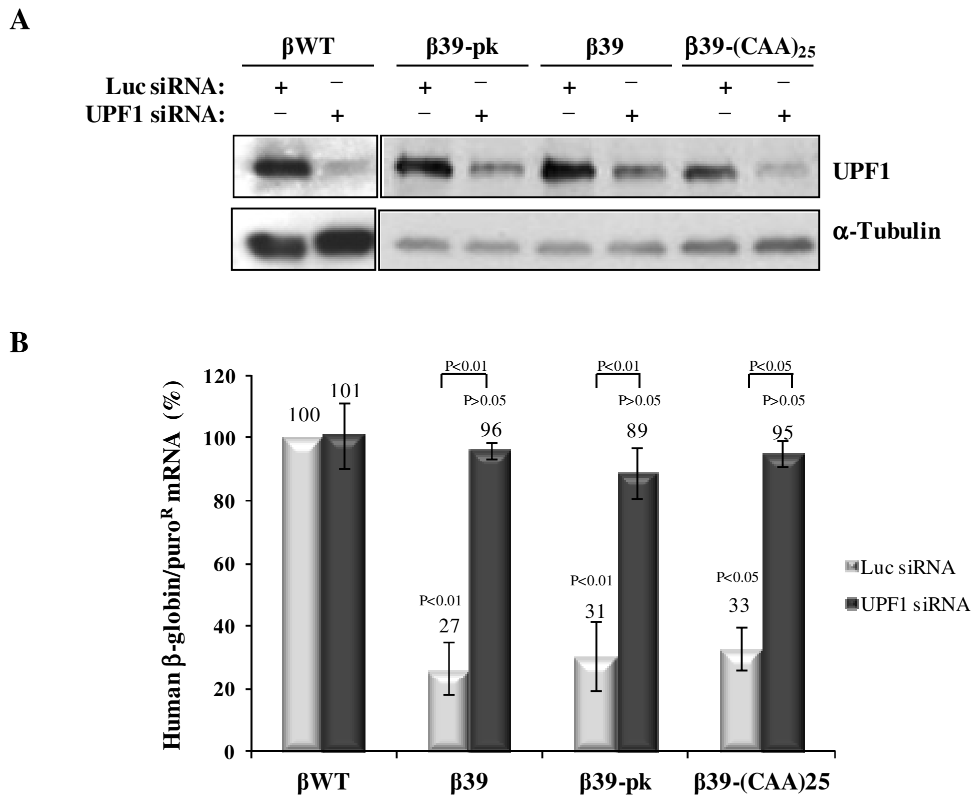

Figure S4. Alterations in the open reading frame (ORF) secondary structure in the context of a long ORF do not affect NMD. (A) Western blot analysis of protein samples obtained from HeLa cells transiently transfected with wild-type (βWT) or with human α-globin constructs nonsense-mutated at codon 39 but containing different ORF structures [β39, β39-pk, β39-$(CAA)_{25}$]. Cells were subjected to single knockdown of UPF1 (UPF1 siRNA) or treated with control siRNA, targeting firefly Luciferase (Luc siRNA). Anti-UPF1 and anti-α-tubulin (control) antibodies were used as indicated. (B) mRNA levels were determined by RT-qPCR using primers specific for human β-globin and puromicin-resistance ($puro^R$) cDNAs. Quantification was performed as above explained in the legend for the supplementary figure S1. Except otherwise indicated, P-values refer to the comparison with the wild-type control transcript levels treated with control siRNA (βWT Luc siRNA).

Table S1. DNA oligonucleotides used in the present work

Primer	Sequence (5' → 3')
#1	CCCACCATGGTGCTGTCTTAGGCCGACAAGACCAACGTC
#2	GACGTTGGTCTTGTCGGCCTAAGACAGCACCATGGTGGG
#3	CAACGTCAAGGCCGCCTAGGGTAAGGTCGGCGCGCAC
#4	GTGCGCGCCGACCTTACCCTAGGCGGCCTTGACGTTG
#5	GTCAAGGCCGCCTGGGGTTAAGTCGGCGCGCACGCTG
#6	CAGCGTGCGCGCCGACTTAACCCCAGGCGGCCTTGAG
#7	GCCAGCGTGCTAGCCGACCTTACC
#8	GGTAAGGTCGGCTAGCACGCTGGC
#9	CCATACTCGCCCTAGTGCGCGCCG
#10	CGGCGCGCACTAGGGCGAGTATGG
#11	GCGCACGCTGGCTAGTATGGTGCGG
#12	CCGCACCATACTAGCCAGCGTGCGC
#13	CACGCTGGCGAGTATTAGGCGGAGGCCCTGGAG
#14	CTCCAGGGCCTCCGCCTAATACTCGCCAGCGTG
#15	GCTGGCGAGTATGGTGCGTAGGCCCTGGAGAGGTGAGG
#16	CCTCACCTCTCCAGGGCCTACGCACCATACTCGCCAGC
#17	GCGGAGGCCCTGTAGAGGTGAGGCT
#18	AGCCTCACCTCTACAGGGCCTCCGC
#19	CTTCTCCCCGCAGGTAGTTCCTGTCCTTCCC
#20	GGGAAGGACAGGAACTACCTGCGGGGAGAAG
#21	CAGGATGTTCCTGTCCTAGCCCACCACCAAGACCT
#22	AGGTCTTGGTGGTGGGCTAGGACAGGAACATCCTG
#23	CTGTCCTTCCCCACCACCTAGACCTACTTCCCGCACTTC
#24	GAAGTGCGGGAAGTAGGTCTAGGTGGTGGGGAAGGACAG
#25	CAAGACCTACTTCCCGTAGTTCGACCTGAGCCACG
#26	CGTGGCTCAGGTCGAACTACGGGAAGTAGGTCTTG
#27	CACTTCGACCTGAGCTAGGGCTCTGCCCAGGT
#28	ACCTGGGCAGAGCCCTAGCTCAGGTCGAAGTG
#29	CACGGCTCTGCCCAGTAGAAGGGCCACGGCAAG
#30	CTTGCCGTGGCCCTTCTACTGGGCAGAGCCGTG
#31	AGGGCCACGGCTAGAAGGTGGCCG
#32	CGGCCACCTTCTAGCCGTGGCCCT
#33	CAAGAAGGTGGCCGACTAGCTGACCAACGCCGTG
#34	CACGGCGTTGGTCAGCTAGTCGGCCACCTTCTTG
#35	GCTGACCAACGCCTAGGCGCACGTGGAC
#36	GTCCACGTGCGCCTAGGCGTTGGTCAGC
#37	GCCGTGGCGCACTAGGACGACATGCCC
#38	GGGCATGTCGTCCTAGTGCGCCACGGC
#39	GCACGTGGACGACTAGCCCAACGCGCTG
#40	CAGCGCGTTGGGCTAGTCGTCCACGTGC
#41	GGACGACATGCCCTAGGCGCTGTCCGCCC
#42	GGGCGGACAGCGCCTAGGGCATGTCGTCC
#43	CGACATGCCCAACGCGTAGTCCGCCCTGAGCGACCTG
#44	CAGGTCGCTCAGGGCGGACTACGCGTTGGGCATGTCG
#45	CCCAACGCGCTGTCCTAGCTGAGCGACCTGCAC

#46	GTGCAGGTCGCTCAGCTAGGACAGCGCGTTGGG
#47	GCGTGCAGGTCCTACAGGGCGGAC
#48	GTCCGCCCTGTAGGACCTGCACGC
#49	CTGTCCGCCCTGAGCGACTAGCACGCGCACAAGCTTC
#50	GAAGCTTGTGCGCGTGCTAGTCGCTCAGGGCGGACAG
#51	CACGCGCACAAGCTTCGGTAAGACCCGGTCAACTTCAAG
#52	CTTGAAGTTGACCGGGTCTTACCGAAGCTTGTGCGCGTG
#53	CCCTCTTCTCTGCACAGCTCTAGAGCCACTGCCTGCTGG
#54	CCAGCAGGCAGTGGCTCTAGAGCTGTGCAGAGAAGAGGG
#55	GCCGCCCACCTCCCCGCCTAGTTCACCCCTGCGGTGCAC
#56	GTGCACCGCAGGGGTGAACTAGGCGGGGAGGTGGGCGGC
#57	CTCCTGCCGACTAGACCAACGTC
#58	GACGTTGGTCTAGTCGGCAGGAG
#59	GACAAGACCAACTAGAAGGCCGCCT
#60	AGGCGGCCTTCTAGTTGGTCTTGTC
#61	CCAACGTCAAGTAGGCCTGGGGTAAG
#62	CTTACCCCAGGCCTACTTGACGTTGG
#63	GAAGGACAGGAACGTCCTGCGGGGAG
#64	CTCCCCGCAGGACGTTCCTGTCCTTC
#65	AGCGCGTTGGGCGTGTCGTCCACG
#66	CGTGGACGACACGCCCAACGCGCT
#67	GTATCAGTCAGGCTCGGCTGGTACCCCTTGCAAAGCGAGCCCACGCT GGCGAGTAT
#68	GTACCAGCCGAGCCTGACTGATACCCCTAGTTGAAGCTTCCATGGTG GGTTCTCTCTG
#69	CTCAGAGAGAACCCACCATGCAACAACAACAACAACAACAACAACA ACAACAACAACAACAACAACAACAACAACAACAACAACAACAACA ACAACAAGAGGCCCTGGAGAGGTGAG
#70	CTCACCTCTCCAGGGCCTCTTGTTGTTGTTGTTGTTGTTGTTGTTGTT GTTGTTGTTGTTGTTGTTGTTGTTGTTGTTGTTGTTGTTGTTGTTG CATGGTGGGTTCTCTCTGAG
#71	CAACAACAACAATAGGCCCTGG
#72	TCTCCAGGGCCTATTGTTGTTG
#73	CCCACCGACTCTAGAGGA
#74	CGCCCACTCAGACTTTATTCA
#75	CCATCGATACATTTGCTTCTGACACAACTG
#76	TTACATGTAGGGATGGGCATAGGCATC
#77	CCCAGGGCCTCACCACCCTATTCATCCACGTTCACC
#78	GGTGAACGTGGATGAATAGGGTGGTGAGGCCCTGGG
#79	CAACCTGCCCAGGGCCTCCTAACCAACTTCATCCACG
#80	CGTGGATGAAGTTGGTTAGGAGGCCCTGGGCAGGTTG
#81	CCAGGGCCTAACCACCAA
#82	TTGGTGGTTAGGCCCTGG
#83	GGTGTCTGTTTGAGGTTGC
#84	ATGGAAGCTTCAACTAGGGGTATCAGTCAGGCTCGGCTGGTACCCCT TGCAAAGCGAGCCGTGGATGAAGTTGGTGGTGAG
#85	ATGGAAGCTTCAACTAGGGGTATCAGTCAGGCTCGGCTGGTACCCCT

	TGCAAAGCGAGCCGTGGATGAATAGGGTGGTGAG
#86	CAACCTCAAACAGACACCATGCAACAACAACAACAACAACAACAAC AACAACAACAACAACAACAACAACAACAACAACAACAACAACAAC AACAAGAGGCCCTGGGCAGGTTG
#87	CAACCTGCCCAGGGCCTCTTGTTGTTGTTGTTGTTGTTGTTGTTGTTG TTGTTGTTGTTGTTGTTGTTGTTGTTGTTGTTGTTGTTGTTGTTGCATG GTGTCTGTTTGAGGTTG
#88	CAACAACAACAATAGGCCCTGGG
#89	TGCCCAGGGCCTATTGTTGTTG
#90	TCCCATTCTAAACTGTACCC
#91	GGAGACAGCACCATGGTGTCTGTTTG
#92	AAACAGACACCATGGTGCTGTCTCC
#93	TCCAGGGCCTCCTCACCACCAACTTCATC
#94	GTTGGTGGTGAGGAGGCCCTGGAGAGG
#95	TCCAGGGCCTCCTAACCACCAACTTCATC
#96	GTTGGTGGTTAGGAGGCCCTGGAGAGGT
#97	TCAGATGCACCATGGTGGGTTCTC
#98	GAGAACCCACCATGGTGCATCTGAC
#99	CAACCTGCCCAGCTCCGCACCATACTCGC
#100	ATGGTGCGGAGCTGGGCAGGTTGGTATCAA
#101	CAACCTGCCCAGCTACGCACCATACTCGCC
#102	ATGGTGCGTAGCTGGGCAGGTTGGTATCAA
#103	GTTTTTGGCGTCTTCCATCCTGCGGGGAGAAG
#104	CTTCTCCCCGCAGGATGGAAGACGCCAAAAAC
#105	CAGAGAGAACCCACCATAGTGCTGTCTCCTGCCGA
#106	TCGGCAGGAGACAGCACTATGGTGGGTTCTCTCTG
#107	GTTTTTGGCGTCTTCTATCCTGCGGGGAGAAG
#108	CTTCTCCCCGCAGGATAGAAGACGCCAAAAAC
#109	GTAGATGAGATGTGACGAACG
#110	GTTTTTGGCGTCTTCCATAACAGCATCAGGAG
#111	CTCCTGATGCTGTTATGGAAGACGCCAAAAAC
#112	CTCAAACAGACACCATAGTGCACCTGACTCCTG
#113	CAGGAGTCAGGTGCACTATGGTGTCTGTTTGAG
#114	CCACTCCTGATGCTGTTATAGAAGACGCCAAAAACATAA
#115	TTATGTTTTTGGCGTCTTCTATAACAGCATCAGGAGTGG
#116	CGACTCTAGAATTACTGCTCG
#117	ATGGAAGACGCCAAAAACATA
#118	GTAAAGCCACCATGGCTTCC
